# Evidence for positive long- and short-term effects of vaccinations against COVID-19 in wearable sensor metrics

**DOI:** 10.1093/pnasnexus/pgad223

**Published:** 2023-07-25

**Authors:** Marc Wiedermann, Annika H Rose, Benjamin F Maier, Jakob J Kolb, David Hinrichs, Dirk Brockmann

**Affiliations:** Computational Epidemiology Group, Robert Koch Institute, 13353 Berlin, Germany; Institute for Theoretical Biology and Integrated Research Institute for the Life-Sciences, Humboldt University of Berlin, 10115 Berlin, Germany; Computational Epidemiology Group, Robert Koch Institute, 13353 Berlin, Germany; Institute for Theoretical Biology and Integrated Research Institute for the Life-Sciences, Humboldt University of Berlin, 10115 Berlin, Germany; Computational Epidemiology Group, Robert Koch Institute, 13353 Berlin, Germany; DTU Compute, Technical University of Denmark, Kongens Lyngby 2800, Denmark; Copenhagen Center for Social Data Science, University of Copenhagen, Copenhagen 1353, Denmark; Computational Epidemiology Group, Robert Koch Institute, 13353 Berlin, Germany; Institute for Theoretical Biology and Integrated Research Institute for the Life-Sciences, Humboldt University of Berlin, 10115 Berlin, Germany; Computational Epidemiology Group, Robert Koch Institute, 13353 Berlin, Germany; Institute for Theoretical Biology and Integrated Research Institute for the Life-Sciences, Humboldt University of Berlin, 10115 Berlin, Germany; Institute for Theoretical Biology and Integrated Research Institute for the Life-Sciences, Humboldt University of Berlin, 10115 Berlin, Germany

**Keywords:** COVID-19, wearable sensors, vaccination, digital health

## Abstract

Vaccines are among the most powerful tools to combat the COVID-19 pandemic. They are highly effective against infection and substantially reduce the risk of severe disease, hospitalization, ICU admission, and death. However, their potential for attenuating long-term changes in personal health and health-related wellbeing after a SARS-CoV-2 infection remains a subject of debate. Such effects can be effectively monitored at the individual level by analyzing physiological data collected by consumer-grade wearable sensors. Here, we investigate changes in resting heart rate, daily physical activity, and sleep duration around a SARS-CoV-2 infection stratified by vaccination status. Data were collected over a period of 2 years in the context of the German Corona Data Donation Project with around 190,000 monthly active participants. Compared to their unvaccinated counterparts, we find that vaccinated individuals, on average, experience smaller changes in their vital data that also return to normal levels more quickly. Likewise, extreme changes in vitals during the acute phase of the disease occur less frequently in vaccinated individuals. Our results solidify evidence that vaccines can mitigate long-term detrimental effects of SARS-CoV-2 infections both in terms of duration and magnitude. Furthermore, they demonstrate the value of large-scale, high-resolution wearable sensor data in public health research.

**Significance Statement**

The increasing use of smartwatches and fitness trackers allows collecting physiological and behavioral data at large scale and high resolution. Here, we show the potential of such data for public health research by tracking vital changes following a SARS-CoV-2 infection in vaccinated and unvaccinated participants of the German Corona Data Donation Project. Daily resting heart rate, activity, and sleep duration showed consistently less average and extreme changes throughout both, the acute phase of COVID-19 and in the long-term, if people were vaccinated prior to infection. Our statistically significant results provide further evidence that vaccinations mitigate (long-term) health constraints of a SARS-CoV-2 infection that can be directly experienced by individuals in their daily lives and are measurable beyond commonly observed clinical outcomes.

## Introduction

COVID-19, the disease caused by infection with SARS-CoV-2, is usually accompanied by, e.g. fever, cough, sore throat, shortness of breath, and fatigue (1). These symptoms are most prevalent during the acute phase of the disease, i.e. the 4 weeks following symptom onset (2), but can persist, develop, or recur for weeks to months (3, 4), a condition known as post-acute sequelae of SARS-CoV-2 infection (PASC) or Long COVID (2, 5–7). Other long-term symptoms include cognitive dysfunction, confusion, chest pain, head- and muscle aches, dizziness, or heart palpitations (8, 9). They can be experienced by all age groups and are not exclusive to people with a severe acute course of the disease (6), though some indication exists that the risk of their contraction can be linked to the number and severity of symptoms experienced at the start of the illness (8).

Vaccines are effective against infection with SARS-CoV-2 as well as hospitalization, ICU admission, and death due to COVID-19 (10–15). However, reports of breakthrough infections after vaccination, especially for the variants of concern B.1.617.2 (Delta) and B.1.1.529 (Omicron), have raised public concern (16). Moreover, despite lowering the risk of symptomatic and severe breakthrough infections (17), it is a subject of contention how well vaccines in such cases also attenuate long-term symptoms potentially associated with PASC/Long COVID (18, 19). Numerous studies report long-term symptoms after breakthrough infections (20–22) with conflicting evidence suggesting that they are either more common (23) or similarly likely than for negative controls (24). In addition, so far only a comparatively low level of certainty exists that the risk of developing prolonged symptoms after breakthrough infections is smaller in comparison to unvaccinated controls (17, 19, 23) as such an effect has only been consistently confirmed for certain sequelae (19, 24, 25).

**Fig. 1.**
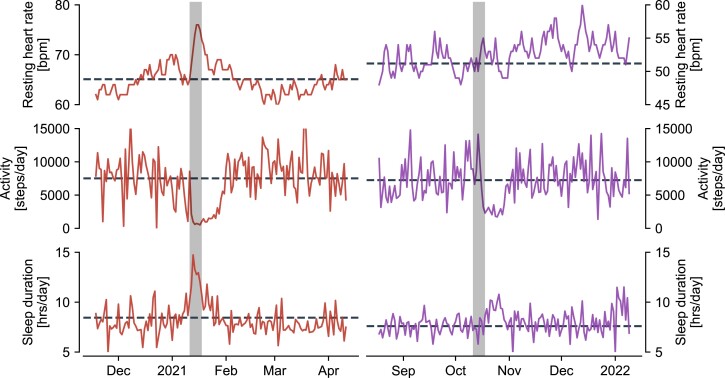
Exemplary time series of daily resting heart rate (top), physical activity (middle), and sleep duration (bottom) for a time window of 150 days in two representative individuals who were unvaccinated (left) and vaccinated (right) at the time of taking a PCR test that returned positive (grey shading). Dashed lines denote the user’s baseline, i.e. the average of the 54 days prior to the test. We observe a strong peak in resting heart rate, a drop in physical activity and increased sleep duration for the unvaccinated individual around the time of the test. Similar patterns are observed for the vaccinated individual with respect to physical activity and sleep duration, the latter change being considerably less pronounced. A visible change of resting heart rate is absent for the vaccinated individual around the week of the test.

Here, we provide evidence that vaccination against SARS-CoV-2 may significantly reduce likelihoods of long-term health constraints after contracting COVID-19. For this purpose, we use large-scale, daily data on resting heart rate (RHR), physical activity, and sleep collected over a period of around 2 years, Fig. 1. These data were collected as part of the Corona Data Donation Project (Corona-Datenspende-App or CDA), a smartphone app that allows users to submit such vital data by linking with consumer-grade smartwatches and fitness trackers. The app was developed at the Robert Koch Institute, Germany’s Federal Public Health Institute and released in Germany on April 12, 2020, to participants of age 16 and older (see Supplementary Material and Fig. S1 for details). As of April 2022, the app had around 190,000 monthly active users with more than 120,000 people submitting daily vital data for at least 600 days. In addition, users optionally participate in in-app surveys about, e.g. diagnostic test results and vaccination data. The high temporal resolution and long observation period permits continuous tracking of an individual’s biometrics and enables a fine-grained analysis of vital signs prior to a SARS-CoV-2 infection, throughout the acute phase of the disease, and during recovery. As such, the combination of survey and vital data enables analysis of long-term physiological and behavioral changes in COVID-19 positive and -negative individuals stratified by vaccination status. We additionally investigate extreme short-term vital changes in the disease’s acute phase as a proxy for symptomatic and severe courses of COVID-19 to show the consistencies between our outcomes, that are measured in a passive fashion while individuals’ go about their everyday activities, with commonly observed clinical factors.

The systematic use of such large-scale data collected via commercially available wearable devices is a growing line of data-driven, medical research (26, 27). It has been applied to study physiological markers of depression (28), characterize daily physiology and circadian rhythms (29), and improve surveillance of influenza-like illnesses (26). In the COVID-19 context, it allowed for early detection of COVID-19 in individuals (30), predicting overall case numbers (31), and discriminating COVID-19 positive from negative individuals (32, 33). Moreover, studies conducted prior to sufficient availability of vaccination data were able to link infections with SARS-CoV-2 to elevated resting heart rate that only returned to baseline levels at an average of 79 days after symptom onset (34).

## Results

We computed weekly changes in resting heart rate (RHR), daily steps, and sleep duration around the date of a COVID-19 PCR test for a total of 8,134 individuals, of which 2,272 experienced a breakthrough infection, 319 were infected prior to receiving their first vaccine dose, and 5,543 reported negative test results, thus serving as a control group (Table 1 and Fig. S2). The per-user baseline against which changes are computed is given by the respective average of each variable over the 8 weeks preceding a confirmed infection with SARS-CoV-2, indicated by the approximate date of a PCR test (given at a weekly temporal resolution). Signals were normalized by subtracting the daily average from all individual time series to account for seasonal effects, e.g. naturally prolonged sleep duration in winter and increased activity in summer. Thus, individual vital data are always measured relative to the population-wide average on a given day. Further details on the characteristics, preprocessing, and analysis of the data are provided in the Materials and Methods Section.

**Table 1.** Number of users per gender as well as mean and standard deviation of age in the respective cohorts under study.

	Vaccinated SARS-CoV-2 positive	Unvaccinated SARS-CoV-2 positive	SARS-CoV-2 negative	Total
Female	915 (40.27%)	132 (41.38%)	2154 (38.86%)	3201 (39.35%)
Male	1350 (59.42%)	185 (57.99%)	3353 (60.49%)	4888 (60.09%)
Other	7 (0.31%)	2 (0.63%)	36 (0.65%)	45 (0.55%)
Age (mean) (years)	48.83	49.69	51.76	50.86
Age (std) (years)	11.35	11.72	12.35	12.13

Percentages in parentheses indicate the respective share of users within the specific cohort. The values for all cohorts are comparable with that of the entire set of participants in the Corona Data Donation project, cf. Table S1.

### Vaccinations mitigate long-term vital changes in COVID-19 positive individuals

**Fig. 2.**
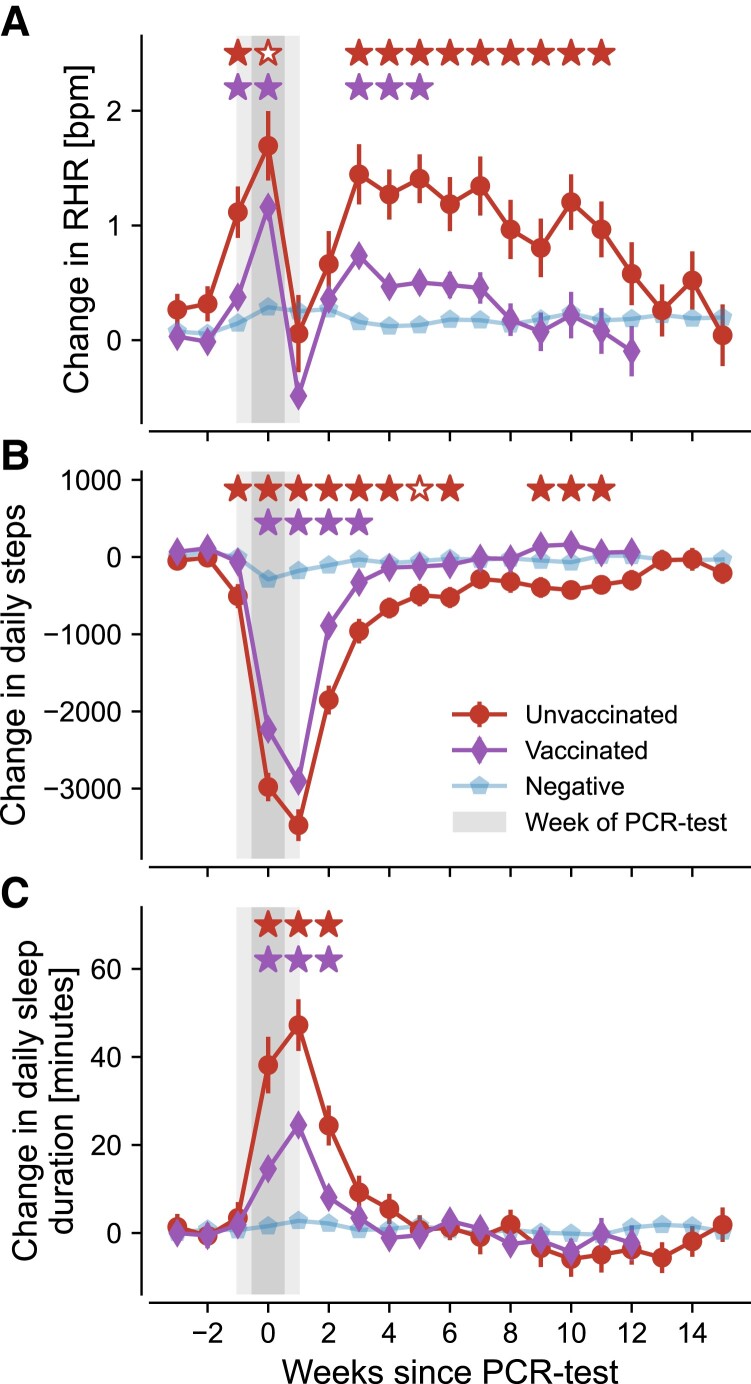
Changes in resting heart rate (RHR), activity, and sleep duration in unvaccinated infected (red circles) and vaccinated infected (purple diamonds) as well as negative controls (blue pentagons). Changes are measured relative to the 2 months preceding the test. Errors bars indicate standard error. Filled red stars (upper row of significance indicators in each panel) indicate that average vital changes of unvaccinated individuals were significantly stronger than for both vaccinated and negative individuals. Hollow red stars indicate that average vital changes of unvaccinated participants were only stronger compared to negative individuals but not compared to the vaccinated cohort. Purple stars (lower row of significance indicators in each panel) indicate significant differences between vaccinated and COVID-19 negative individuals. All three assessments use a one-sided Welch *t*-test with a significance level of α=0.01. Grey shading indicates the uncertainty in estimating the precise timing of a PCR test, since users only submit this information in terms of the respective calendar week. Results for the vaccinated infected cohort (purple diamonds) are only shown for the first 12 weeks after a positive PCR test due to insufficient data for later time periods. Individuals are considered part of the vaccinated cohort when they received at least two doses of mRNA vaccine.

We first evaluated the evolution of average changes in weekly RHR, step count, and sleep duration in the weeks following a PCR test separately for each user cohort, depicted in Fig. 2.

On average, the RHR of unvaccinated users with SARS-CoV-2 infection increased by ∼1.7 beats per minute (bpm) in the week of the PCR test and only returned to baseline levels after 11 weeks (Fig. 2A). This finding qualitatively confirms similar results obtained in earlier studies (34, 35) that did not specifically differentiate by vaccination status. We found a pronounced drop in RHR around 1 week after a PCR test, with values that decreased even below baseline for vaccinated users. This aligns with earlier studies (34) and potentially indicates transient bradycardia following infection (36). RHR of unvaccinated individuals already increased significantly in the week preceding a PCR test at values of ∼1.1 bpm above normal. We found weaker average deviations in RHR for vaccinated individuals with a maximum value of ∼1.2 bpm in the week of the PCR test. These deviations were accompanied by a swifter return to baseline levels after approximately 3–6 weeks. Except for the 2 weeks following a PCR test, the average RHR-change for vaccinated individuals was approximately two to three times lower than for those that were unvaccinated (Fig. 2A), potentially indicating a milder course of the disease on average.

The average daily activity (Fig. 2B) decreased in the week of the PCR test by ∼2,000 and ∼3,000 steps per day for vaccinated and unvaccinated individuals, respectively. For both groups, the reduction usually began the week of a positive PCR test and thus might have been partially modulated by changes in behavior, i.e. self-isolation. A return to baseline activity among vaccinated individuals occurred after only 4 weeks compared to around 6–11 weeks for the unvaccinated cohort, indicating prolonged alteration in the average activity after an infection with SARS-CoV-2 for the latter group.

Finally, the average sleep duration of unvaccinated individuals increased abruptly by ∼37 minutes per day during the week of the PCR test. For vaccinated individuals, this effect was reduced by more than half to an average of only ∼15 minutes per day. Similar magnitudes were observed in the first week following a PCR test, where sleep duration was increased by ∼46 and ∼24 minutes per day for unvaccinated and vaccinated users, respectively. Sleep duration returned to baseline values more quickly for both user groups as compared to activity or RHR. By the third week, anomalies in sleep duration were comparable with that of the COVID-19 negative control group. Still, we found a significant increase of approximately two times in the average need for rest during the acute phase of the disease when comparing vaccinated and unvaccinated individuals.

We also found small variations in RHR, activity, and sleep duration in COVID-19 negative individuals around the test period, potentially related to other diseases, such as influenza or the common cold, which might have caused people to take a PCR test in the first place.

### Extreme changes in acute phase most prominent for unvaccinated individuals

**Fig. 3.**
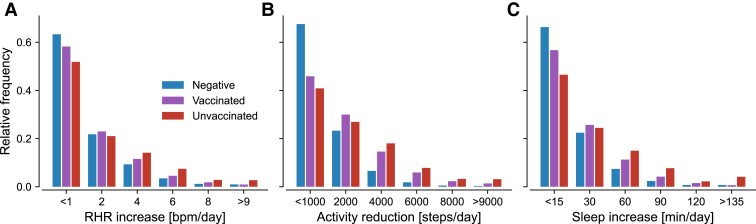
Relative frequency of average vital changes in weeks 0 to 4 following a PCR test for vaccinated and unvaccinated COVID-19 positive as well as COVID-19 negative individuals. Numbers on the vertical axis indicate the center of each bin, except for the left- and right-most bins that include all data smaller or larger than the given values, respectively. Individuals are considered part of the vaccinated cohort when they received at least two doses of mRNA vaccine.

In addition to the observed prolonged changes in vitals, the most prominent signals appeared during the acute phase of the disease, cf. Fig. 2. For a thorough assessment, we therefore also investigated the distributions of weekly increases in RHR and sleep duration as well as decreases in activity for each cohort in the 4 weeks following a PCR test, Fig. 3.

For all metrics, the frequencies of changes decayed continuously with increasing values. As expected, the smallest changes (less than 1 bpm/day of RHR change, less than 1,000 steps/day reduction in activity, and less than 15 min/day of additional sleep) were most commonly observed in the COVID-19 negative cohort. Likewise, between 40% and 50% of all COVID-19 positive individuals experienced only small changes in all three metrics, likely indicating comparatively mild courses. Note that these numbers are well below the commonly reported percentage of mild and moderate cases of at least 80% (37) while exceeding rough estimates (∼41%) for asymptomatic infections in confirmed COVID-19 cases (38). Hence, we found a reasonable amount of individuals with little or no changes in their vital data during SARS-CoV-2 infection. The observed frequencies of larger and more extreme vital changes in the unvaccinated cohort were consistently higher than those measured for vaccinated and COVID-19 negative individuals, again indicating increased likelihood for a severe course in unvaccinated individuals, Fig. 3.

### Vaccinations reduce short-term risk of severe vital changes in COVID-19 positive individuals

**Fig. 4.**
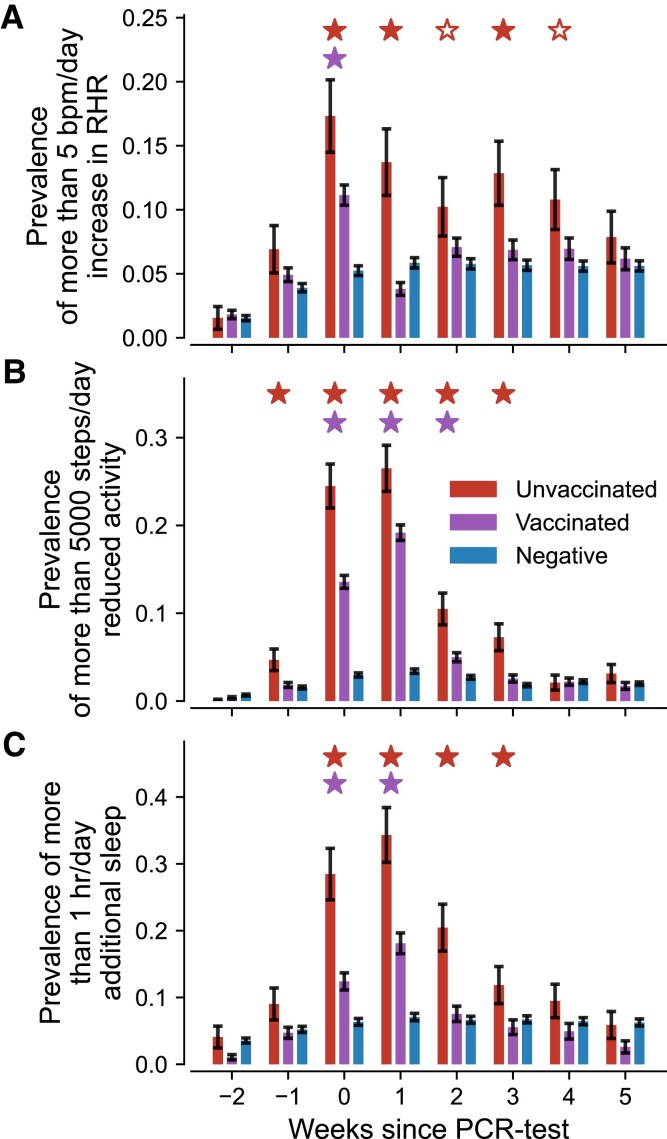
Share of individuals in each cohort whose weekly average vital changes exceeded a specified threshold. A) Share of individuals with more than 5 bpm/day RHR increase. B) Share of individuals with an activity reduction of more than 5,000 steps/day. C) Share of individuals with an increased sleep duration of more than 1 h/day. Red (left), purple (center), and blue (right) bars indicate weekly prevalences for the unvaccinated, vaccinated, and COVID-19 negative cohort, respectively. Error bars indicate the standard error of a binomial distribution. Filled red stars (upper row of significance indicators in each panel) indicate periods where the prevalences in unvaccinated individuals were stronger than for both vaccinated and negative individuals. Hollow red stars indicate periods where the prevalences in unvaccinated individuals were only stronger than that of negative individuals but not of the vaccinated cohort. Purple stars (lower row of significance indicators in each panel) indicate significant differences between vaccinated and COVID-19 negative individuals. All three assessments use a one-sided two proportion *z*-test with a significance level of α=0.01. Individuals are considered part of the vaccinated cohort when they received at least two doses of mRNA vaccine.

In order to clarify the prevalence of severe courses in acute cases, we ultimately measured how extreme changes in vital signals were distributed for the unvaccinated, vaccinated, and COVID-19 negative cohorts in the first weeks after taking a PCR test, Fig. 4. For this purpose, we computed within each cohort the share of users whose vital changes exceeded a certain threshold. Specifically, we counted individuals whose change in RHR exceeded 5 bpm/day and define an extreme activity reduction of 5,000 steps/day as well as pronounced sleep prolongation of more than 1 hr/day, both of which well exceed the maximum observed average change in Fig. 2 while still yielding reasonable frequencies in Fig. 3.

In the unvaccinated group, the frequency of more than 5 bpm/day RHR change varied between 17.5% in the week of and 10% in the fourth week after a positive PCR test, Fig. 4A. For weeks 0, 1, and 3 those frequencies were significantly larger than those of the vaccinated cohort. The same held true for weeks 2 and 4 when comparing the unvaccinated cohort with the COVID-19 negative control group. Only from week five onward were extreme RHR changes equally likely in COVID-19 positive individuals as in the negative control cohort. In contrast, extreme RHR changes in the vaccinated cohort were only significantly more frequent than for negative controls during the week of the PCR test while being only half as common than in the unvaccinated cohort.

Likewise, we found significantly increased prevalence of drastic reduction in activity for both the vaccinated and unvaccinated cohorts in the first 2–3 weeks following a PCR test when compared to the COVID-19 negative control group, Fig. 4B. In addition, the respective prevalence in the unvaccinated cohort was significantly larger compared to the vaccinated group in weeks -1 to 3, again indicating a reduced risk of severe illness following full vaccination. Notably, a small share (∼5%) of unvaccinated individuals already showed substantial activity reduction in the week prior to taking a PCR test, indicating a potential precursor for the developing disease. No significant extreme reductions in activity were observed after the third week, Fig. 4B.

Finally, we considered the frequency of individuals with an increased sleep duration of 1 hr/day, indicative of a strongly increased need for rest in the acute phase of COVID-19, Fig. 4C. Both COVID-19 positive cohorts showed greatly increased frequency in sleep prolongation during weeks 0 and 1 following a PCR test, with more than 30% of cases in the unvaccinated group and 10–20% of cases in the vaccinated cohort. Extended sleep duration was also significantly prominent in the unvaccinated cohort in the second week after a positive PCR test with a prevalence of more than 20% compared to less than 10% in the vaccinated and the control cohorts. Hence, roughly 3–4 out of 10 unvaccinated individuals experienced an increased sleep duration of more than 1 hr/day for an extended period of 2–3 weeks.

## Discussion

We analyzed changes in resting heart rate (RHR), physical activity, and sleep duration around the time of a PCR test for 2,272 vaccinated and 319 unvaccinated COVID-19 positive individuals as well as 5,543 individuals in a COVID-19 negative control group. Participants in this study were self-recruited, often following media announcements. They submitted their vital data and meta-information, i.e. sociodemographics, PCR test dates and results, and vaccination status, via the Robert Koch Institute’s Corona-Datenspende smartphone app (CDA), downloadable free of charge for German residents over the age of 16.

We found that average deviations and subsequent stabilizations in vital signals were most pronounced for unvaccinated individuals, with the longest normalization period spanning an average of 11 weeks post-PCR test week for both RHR and activity. Similar findings have been obtained in other studies that, although not explicitly stated, likely mostly considered unvaccinated individuals due to the scarcity of vaccines at the time (34, 35). Average vital changes for vaccinated persons were less pronounced, albeit at times still significantly different from the COVID-19 negative control group. In addition, extreme values were more likely observed for unvaccinated individuals in the acute phase of the disease when compared to the vaccinated/negative cohort, indicating the consistency with common clinical outcomes for estimating vaccine efficacies against severe and symptomatic cases of COVID-19 (10, 11, 13, 15). Finally, we observed that both RHR as well as the step count of unvaccinated COVID-19 positive individuals, already differed significantly from the negative control group in the week prior to taking a PCR test, hinting at its potential to serve as an early warning indicator of a coming illness (26, 30). Our results provide further evidence that vaccinations can not only mitigate severe cases of acute COVID-19, which is in line with the broader literature (10–14, 17, 39) but also highlight their potential for attenuating long-term physiological and behavioral changes (18, 20, 22, 23, 25). Our results exemplify the great potential that lies in passive sensing for public health research as it fosters robust and large-scale analysis of long-term, high-resolution longitudinal data with interpretable metrics that can be linked to an individual’s physiology (40). As such, it can provide an additional proxy for individual health-related wellbeing at the time of measurement in terms of, e.g., activity and sleep patterns, and thus serve as a complement to common clinical and diagnostic outcomes.

Our analysis comes with some limitations that need to be considered when contextualizing the above results. First and foremost, our analysis did not discriminate infections by the respective variant of concern (VOC) that was predominant at the time a PCR test was taken. Furthermore, individuals that were designated as unvaccinated were primarily assigned to this cohort because, at the time of infection, vaccine availability was limited (see Supplementary Material and Fig. S2). Hence, all unvaccinated donors were likely infected with B.1.1.7 (Alpha) or the wild-type which might have triggered different physiological responses than the later emergent variants, specifically B.1.617.2 (Delta) and, more recently, B.1.1.529 (Omicron). The majority of breakthrough infections in our data set were reported when these latter two variants were prevalent, meaning that the observed effects could partially also be explained by weaker physiological responses to Omicron (41). To account for this effect, we performed an additional sensitivity analysis (see Supplementary Material) that only considered infections reported prior to 2021 December 15. These breakthrough infections are then mostly recorded for the forth wave of the pandemic (42) and thus likely caused by Delta, which has been reported to cause more severe cases than Alpha (43). When only considering this subset of users in the vaccinated cohort, we found that the results presented in Fig. 2 still hold, indicating that vaccinated individuals likely infected with the Delta variant exhibited significantly weaker average changes in vital data compared to the unvaccinated group, Fig. S3. We also compared average vital changes in that same cohort of vaccinated individuals infected before 2021 December 15 to individuals who reported infections after that date, i.e. during the fifth wave of the pandemic in Germany. The latter were thus likely infected with the Omicron VOC. We found hardly any significant differences in the temporal evolution of vital changes between the cohorts, Fig. S4. Hence, in the context of our analysis, it is reasonable to combine all recorded breakthrough infections into a single cohort for ease of interpretability and without having results skewed by the influence of a single variant of concern.

By now, almost all users of the Corona Data Donation Project are at least fully vaccinated or have received a booster vaccination. Physiological responses to more recent variants of concern in unvaccinated individuals could, therefore, only be recorded if unvaccinated individuals were specifically recruited. In addition, we did not explicitly account for the time between receiving the latest vaccination dose and the date of breakthrough infection, which ignores the potential effects of waning immunity (44). However, most breakthrough infections in our data set were recorded in the first 4 months after receiving the last of at least two vaccine doses (see Supplementary Material and Fig. S5), likely indicating consistent protection in the majority of the vaccinated cohort.

We also note that neither of our three cohorts is representative of the German population. Our sample shows a large over-representation of male individuals (see Table 1), as well as an under-representation of adolescent and elderly (65+) persons, see Supplementary Material and Fig. S6 for the distribution of age groups. Moreover, there is good reason to assume that our study population is more health conscious than the average population since the usage of fitness trackers is partially correlated with or, at least, facilitates awareness of health-related behavior (45). Likewise, the cohorts might not be fully representative of one another, even though the basic proportions of gender and age match well across them, Table 1. In addition, the distributions of age and gender are consistent between our study subsample and the entire set of participants in the Corona Data Donation project (see Fig. S6 and Table S1), indicating that there is at least no additional selection bias for partaking in the surveys about test results and vaccination dates. We note that there are many more confounding factors that might influence the observed vital changes, such as pre-existing conditions, self-reported symptoms during the disease, socioeconomic status, as well as demographics including age, sex, and body mass index. Due to the limited sample size, we refrained from performing any further discrimination along these potentially confounding factors, but such analyses could be performed in the future if the recorded cohorts increased in size. However, despite the above limitations, our analysis provides relevant insights regarding the efficacy of vaccine against long-term effects of COVID-19. Because vulnerable groups, such as the elderly, are under-represented, the observed differences might become more pronounced if more people from such groups participated in the study. As such, the results for unvaccinated individuals might indicate a lower bound for expected vital changes which might become larger when a more representative cohort is considered, thereby potentially increasing the observed differences between our cohorts further.

Furthermore, we acknowledge that we cannot disentangle whether changes in vital data, especially activity and sleep duration, were caused by a behavioral response to a positive diagnosis or whether those changes were an actual physiological imprint of an acute infection. While we likely observed a combination of both effects, it remains impossible to quantify their individual influence. However, we may assume that the mere effect of isolation reduces the opportunity for physical activity equally for unvaccinated and vaccinated individuals, thereby making it an unlikely explanation for all the observed changes in daily activity. Still, across all metrics, the average deviation in step counts were most similar between the COVID-19 positive cohorts, indicating that these changes are, in fact, partially driven by self-isolation. In order to better understand these effects, future studies of similar kind should specifically aim to survey individual behavior after an infection, including whether and for how long participants went into self-isolation. Along the same lines, additional information on actual symptoms, such as fever or fatigue, could serve to explain magnitudes and duration of the observed vital changes and allow to further discriminate the considered cohorts in a meaningful manner for an even more fine-grained analysis.

Future work should aim to reproduce and validate the results obtained in this study, ideally with data that are collected in a similar fashion such as through the *DETECT* (46) or *Evidation* (47) systems in the US, as well as *TemPredict* which covers a broad range of international users (48). We further propose to investigate and improve the representativeness of the user sample, i.e. by comparison with common health survey programs, such as GEDA in Germany (49) or NHIS in the US (50). One should then aim to specifically advertise for an increased participation of currently under-represented groups, potentially by providing wearables to users that could normally not afford such devices and are therefore missing from the data set. Moreover, one should aim to collect data, such as respiratory rate, body temperature, or even oxygen saturation (especially SpO2) with a potentially more direct indication of impending illness as well as the severity of symptoms in the acute phase and the long-term. This also carries the potential for future clinical applications and individual-level monitoring, e.g., to indicate when patients should seek medical care or to monitor recovery and returning physical resilience after a disease. While the present analysis investigates the potential magnitude and duration of long-term vital changes as well as the prevalence of certain extreme signals in the acute phase, we suggest that future work should aim for a combined analysis of the two effects with the goal of assessing potential similarities between them on the individual level. This would allow to further identify to what extent the persistence of lingering symptoms can be understood as a potential reflection of a severe acute phase. Ultimately, we suggest to incorporate higher-frequency data recorded with a temporal resolution on the scale of minutes (29). Such data would allow for the quantification of more subtle changes in physiology, such as those observed in postural orthostatic tachycardia syndrome (POTS), another typical condition associated with Long COVID (51). After all, it is a unique advantage of wearable sensors that data can be measured over extended periods at high resolution and minimal burden to the individual, thereby making them a promising tool to complement traditional clinical methods for a data-driven approach to public health research (46, 52).

## Materials and methods

### Data characteristics

Between April 12, 2020, and April 3, 2022, a total of 535,557 people installed the Corona-Datenspende App and submitted at least one vital data point. Of these users, 48,281 people agreed to also participate in regular surveys about COVID-19 test results, vaccination status, and other information relevant for pandemic research. 29,486 users submitted their vaccination status and the months of receiving doses, and 20,614 provided the week and result of their first positive PCR test or their first ever PCR test if all results were negative. The overlap between users who submitted both vaccination status and test results resulted in 16,928 people.

### Data preprocessing

Due to inconsistent measurements in sleep duration for Apple devices following a manufacturer update on October 10, 2022, 532 affected users were removed from the data set. In addition, 341 users who received a single dose with the vaccine Ad26.COV2.S (Janssen) and 40 users who were infected after receiving their first mRNA vaccine dose were excluded from the analysis. Of this subset, we kept users who donated at least one vital data point between 8 weeks preceding and 20 weeks following their PCR test (12,308 users). We computed weekly averages if at least six data points were present in a given week. Users with less than 3 weeks of sufficient data preceding their test were dropped since no reliable baseline could be computed, leaving a total of 8,134 users. We considered all users who received at least two vaccination doses prior to their positive PCR test as *vaccinated* and those who did not receive any dose as *unvaccinated*, such that 2,272 individuals experienced a breakthrough infection, 319 were infected prior to receiving their first vaccine dose, and 5,543 only reported negative PCR tests. A detailed cohort-diagram can be found in Fig. S7.

### Data analysis

As a first step, we computed per-user anomalies of all vital data. For this purpose, we subtracted daily population-wide averages of resting heart rate, step count, and sleep duration from each user’s time series in order to account for seasonal effects, such as increased activity in summer or prolonged sleep duration in winter. For an increased accuracy, we used the data of all 16,396 users that are left in the study cohort after removing Apple users with inconsistent data for this purpose. After this transformation, a value of zero indicated that a user’s vital measurement was en par with the population-wide average on a given day, while positive and negative values indicated above- and below-average values, respectively. A visualization of these preprocessing steps by means of transforming an exemplary vital time series is shown in Fig. S8.

Next, user data were down-sampled into weekly bins, enabling the same temporal resolution as the approximate date of reported PCR tests. We then computed for each user and vital metric an individual baseline as the average over the 8 weeks prior to the PCR test. We subtracted this baseline from each time series to obtain the corresponding deviations in vital signals. For all users, the time series were then aligned with the week of a PCR test and averaged (for the results in Fig. 2) or thresholded (for the results in Fig. 4) depending on the desired analysis.

### Statistical analysis

For all discussions of differences in average vitals, we used a one-sided Welch *t*-test. For analyzing the prevalence of extreme vital changes, we applied a one-sided two proportion *z*-test. For both tests, we used a significance level of α=0.01. One-sided tests were used since we put our focus on whether vital changes of unvaccinated individuals *exceeded* those of the vaccinated or negative cohorts. Likewise, we were only interested in vital changes of vaccinated individuals if they *exceeded* those measures observed for the negative control cohort. We evaluated quantile–quantile diagrams to compare observed vital changes with those expected from a normal distribution. The quantiles show a strong linear relationship (Figs. S9–S17) with only a few exceptions of slightly skewed data that is very well within the limits of what can be tolerated by a *t*-test. Hence, we may consider our data to be sufficiently normally distributed for statistical analysis. We also ensured the absence of (a larger set of) prominent outliers that might artificially skew any of the depicted averages in Fig. 2 toward increased or decreased values (see Fig. S18).

## Supplementary Material

pgad223_Supplementary_Data

## Data Availability

The data analyzed in this study concerns the health condition of individual persons. As per the General Data Protection Regulation (GDPR) such data constitutes sensitive information and is protected as a “special category of personal data.” For this reason, the data can not be directly shared in a public repository, but interested parties can request access to the data following registration with the Data Privacy Officer at the Robert Koch-Institute. All initial data inquiries should be addressed to Marc Wiedermann (wiedermannm@rki.de) or the general contact address of the Corona Data Donation project (corona-datenspende@rki.de).

## References

[1] Singhal T . 2020. A review of coronavirus disease-2019 (COVID-19). Indian J Pediatr. 87:281–286. 32166607 10.1007/s12098-020-03263-6 PMC7090728

[2] Nalbandian A , *et al*. 2021. Post-acute COVID-19 syndrome. Nat Med. 27:601–615. 33753937 10.1038/s41591-021-01283-z PMC8893149

[3] Wynberg E , *et al*. 2021. Evolution of coronavirus disease 2019 (COVID-19) symptoms during the first 12 months after illness onset. Clin Infect Dis. 75:ciab759. 10.1093/cid/ciab759 PMC8522402 34473245

[4] Michelen M , *et al*. 2021. Characterising long COVID: a living systematic review. BMJ Global Health. 6:e005427. 10.1136/bmjgh-2021-005427 PMC8478580 34580069

[5] Soriano JB , Murthy S, Marshall JC, Relan P, Diaz JV. 2021. A clinical case definition of post-COVID-19 condition by a Delphi consensus. Lancet Infect Dis. 22:e102–e107. 34951953 10.1016/S1473-3099(21)00703-9 PMC8691845

[6] Alwan NA . 2021. The road to addressing long COVID. Science. 373:491–493. 34326224 10.1126/science.abg7113

[7] Huang C , *et al*. 2021. 6-month consequences of COVID-19 in patients discharged from hospital: a cohort study. Lancet. 397:220–232. 33428867 10.1016/S0140-6736(20)32656-8 PMC7833295

[8] Ziauddeen N , *et al*. 2022. Characteristics and impact of long covid: findings from an online survey. PLoS One. 17:e0264331. 35259179 10.1371/journal.pone.0264331 PMC8903286

[9] Sudre CH , *et al*. 2021. Attributes and predictors of long COVID. Nat Med. 27:626–631. 33692530 10.1038/s41591-021-01292-y PMC7611399

[10] Tregoning JS , Flight KE, Higham SL, Wang Z, Pierce BF. 2021. Progress of the COVID-19 vaccine effort: viruses, vaccines and variants versus efficacy, effectiveness and escape. Nat Rev Immunol. 21:626–636. 34373623 10.1038/s41577-021-00592-1 PMC8351583

[11] Higdon MM , *et al*. 2022. A systematic review of COVID-19 vaccine efficacy and effectiveness against SARS-CoV-2 infection and disease. medRxiv.

[12] Bahl A , *et al*. 2021. Vaccination reduces need for emergency care in breakthrough COVID-19 infections: a multicenter cohort study. Lancet Reg Health - Am. 4:100065. 34522911 10.1016/j.lana.2021.100065 PMC8428472

[13] Butt AA , *et al*. 2021. Outcomes among patients with breakthrough SARS-CoV-2 infection after vaccination. Int J Infect Dis. 110:353–358. 34375762 10.1016/j.ijid.2021.08.008 PMC8349447

[14] Cabezas C , *et al*. 2021. Associations of BNT162b2 vaccination with SARS-CoV-2 infection and hospital admission and death with covid-19 in nursing homes and healthcare workers in Catalonia: prospective cohort study. BMJ. 374:n1868. 34407952 10.1136/bmj.n1868 PMC8371258

[15] Glatman-Freedman A , *et al*. 2021. Effectiveness of BNT162b2 vaccine in adolescents during outbreak of SARS-CoV-2 delta variant infection, Israel, 2021. Emerging Infect Dis. 27:2919–2922. 10.3201/eid2711.211886 PMC8544958 34570694

[16] Nixon DF , Ndhlovu LC. 2021. Vaccine breakthrough infections with SARS-CoV-2 variants. N Engl J Med. 385:e7. 10.1056/NEJMc2107808 34077640

[17] Antonelli M , *et al*. 2022. Risk factors and disease profile of post-vaccination SARS-CoV-2 infection in UK users of the COVID Symptom Study app: a prospective, community-based, nested, case-control study. Lancet Infect Dis. 22:43–55. 34480857 10.1016/S1473-3099(21)00460-6 PMC8409907

[18] Ledford H . 2021. Do vaccines protect against long COVID? What the data say. Nature. 599:546–548. 34815580 10.1038/d41586-021-03495-2

[19] Notarte KI , *et al*. 2022. Impact of COVID-19 vaccination on the risk of developing long-covid and on existing long-covid symptoms: a systematic review. EClinicalMedicine. 53:101624. 36051247 10.1016/j.eclinm.2022.101624 PMC9417563

[20] Massey D , Berrent D, Krumholz H. 2021. Breakthrough symptomatic COVID-19 infections leading to long covid: report from long covid facebook group poll. medRxiv. 10.1101/2021.07.23.21261030.

[21] Bergwerk M , *et al*. 2021. Covid-19 breakthrough infections in vaccinated health care workers. N Engl J Med. 385:1474–1484. 34320281 10.1056/NEJMoa2109072 PMC8362591

[22] Stephenson T , *et al*. 2022. Long COVID - the physical and mental health of children and non-hospitalised young people 3 months after SARS-CoV-2 infection; a national matched cohort study (The CLoCk) study. Res Sq.

[23] Al-Aly Z , Bowe B, Xie Y. 2022. Long COVID after breakthrough SARS-CoV-2 infection. Nat Med. 28:1461–1467. 35614233 10.1038/s41591-022-01840-0 PMC9307472

[24] Kuodi P , *et al*. 2022. Association between BNT162b2 vaccination and reported incidence of post-COVID-19 symptoms: cross-sectional study 2020–21, Israel. NPJ Vaccines. 7:1–8. 36028498 10.1038/s41541-022-00526-5 PMC9411827

[25] Taquet M , Dercon Q, Harrison PJ. 2021. Six-month sequelae of post-vaccination SARS-CoV-2 infection: a retrospective cohort study of 10,024 breakthrough infections. medRxiv. 10.1101/2021.10.26.21265508.

[26] Radin JM , Wineinger NE, Topol EJ, Steinhubl SR. 2020. Harnessing wearable device data to improve state-level real-time surveillance of influenza-like illness in the USA: a population-based study. Lancet Digit Health. 2:e85–e93. 33334565 10.1016/S2589-7500(19)30222-5 PMC8048388

[27] Colombo D , *et al*. 2019. Current state and future directions of technology-based ecological momentary assessment and intervention for major depressive disorder: a systematic review. J Clin Med. 8:465. 30959828 10.3390/jcm8040465 PMC6518287

[28] Ghandeharioun A , *et al*. 2017. Objective assessment of depressive symptoms with machine learning and wearable sensors data. 2017 Seventh International Conference on Affective Computing and Intelligent Interaction (ACII). p. 325–332. San Antonio, TX, USA.

[29] Bowman C , *et al*. 2021. A method for characterizing daily physiology from widely used wearables. Cell Reports Methods. 1:100058. 34568865 10.1016/j.crmeth.2021.100058 PMC8462795

[30] Mishra T , *et al*. 2020. Early detection Of COVID-19 using a smartwatch. medRxiv. 10.1101/2020.07.06.20147512.

[31] Zhu G , *et al*. 2020. Learning from large-scale wearable device data for predicting the epidemic trend of COVID-19. Discrete Dyn Nat Soc. 2020:e6152041.

[32] Quer G , *et al*. 2021. Wearable sensor data and self-reported symptoms for COVID-19 detection. Nat Med. 27:73–77. 33122860 10.1038/s41591-020-1123-x

[33] Gadaleta M , *et al*. 2021. Passive detection of COVID-19 with wearable sensors and explainable machine learning algorithms. npj Digit Med. 4:1–10. 34880366 10.1038/s41746-021-00533-1 PMC8655005

[34] Radin JM , *et al*. 2021. Assessment of prolonged physiological and behavioral changes associated with COVID-19 infection. JAMA Netw Open. 4:e2115959. 34232306 10.1001/jamanetworkopen.2021.15959 PMC8264646

[35] Natarajan A , Su HW, Heneghan C. 2022. Occurrence of relative bradycardia and relative tachycardia in individuals diagnosed with COVID-19. medRxiv. 10.1101/2022.02.02.22270342.

[36] Amaratunga EA , Corwin DS, Moran L, Snyder R. 2020. Bradycardia in patients with COVID-19: a calm before the storm? Cureus. 12:e8599. 32550090 10.7759/cureus.8599 PMC7294893

[37] Wu Z , McGoogan JM. 2020. Characteristics of and important lessons from the coronavirus disease 2019 (COVID-19) outbreak in China: summary of a report of 72,314 cases from the Chinese center for disease control and prevention. JAMA. 323:1239–1242. 32091533 10.1001/jama.2020.2648

[38] Ma Q , *et al*. 2021. Global percentage of asymptomatic SARS-CoV-2 infections among the tested population and individuals with confirmed COVID-19 diagnosis: a systematic review and meta-analysis. JAMA Netw Open. 4:e2137257. 34905008 10.1001/jamanetworkopen.2021.37257 PMC8672238

[39] Abu-Raddad LJ , Chemaitelly H, Butt AA. 2021. Effectiveness of the BNT162b2 COVID-19 vaccine against the B.1.1.7 and B.1.351 variants. N Engl J Med. 385:187–189. 33951357 10.1056/NEJMc2104974 PMC8117967

[40] Cornet VP , Holden RJ. 2018. Systematic review of smartphone-based passive sensing for health and wellbeing. J Biomed Inform. 77:120–132. 29248628 10.1016/j.jbi.2017.12.008 PMC5793918

[41] Chen J , Wang R, Gilby NB, Wei GW. 2022. Omicron variant (B.1.1.529): infectivity, vaccine breakthrough, and antibody resistance. J Chem Inf Model. 62:412–422. 34989238 10.1021/acs.jcim.1c01451 PMC8751645

[42] Maier BF , *et al*. 2021. Germany’s current COVID-19 crisis is mainly driven by the unvaccinated. medRxiv. 10.1101/2021.11.24.21266831.

[43] Sheikh A , McMenamin J, Taylor B, Robertson C. 2021. SARS-CoV-2 Delta VOC in Scotland: demographics, risk of hospital admission, and vaccine effectiveness. Lancet. 397:2461–2462. 34139198 10.1016/S0140-6736(21)01358-1 PMC8201647

[44] Tartof SY , *et al*. 2021. Effectiveness of mRNA BNT162b2 COVID-19 vaccine up to 6 months in a large integrated health system in the USA: a retrospective cohort study. Lancet. 398:1407–1416. 34619098 10.1016/S0140-6736(21)02183-8 PMC8489881

[45] Wu Q , Sum K, Nathan-Roberts D. 2016. How fitness trackers facilitate health behavior change. Proc Hum Factors Ergon Soc Annu Meet. 60:1068–1072.

[46] Radin JM , Quer G, Jalili M, Hamideh D, Steinhubl SR. 2021. The hopes and hazards of using personal health technologies in the diagnosis and prognosis of infections. Lancet Digit Health. 3:e455–e461. 34020933 10.1016/S2589-7500(21)00064-9

[47] Shapiro A , *et al*. 2021. Characterizing COVID-19 and influenza illnesses in the real world via person-generated health data. Patterns. 2:100188. 33506230 10.1016/j.patter.2020.100188 PMC7815963

[48] Mason AE , *et al*. 2022. Detection of COVID-19 using multimodal data from a wearable device: results from the first TemPredict Study. Sci Rep. 12:3463. 35236896 10.1038/s41598-022-07314-0 PMC8891385

[49] Damerow S , *et al*. 2020. Developments in the health situation in Germany during the initial stage of the COVID-19 pandemic for selected indicators of GEDA 2019/2020-EHIS. J Health Monit. 5:3–20. 10.25646/7172.2 PMC8823006 35146276

[50] National Center for Health Statistics (US) . 1986. Division of Health Interview Statistics, National Health Interview Survey.

[51] Miglis MG , *et al*. 2020. A case report of postural tachycardia syndrome after COVID-19. Clin Auton Res. 30:449–451. 32880754 10.1007/s10286-020-00727-9 PMC7471493

[52] Trifan A , Oliveira M, Oliveira JL. 2019. Passive sensing of health outcomes through smartphones: systematic review of current solutions and possible limitations. JMIR Mhealth Uhealth. 7:e12649. 31444874 10.2196/12649 PMC6729117

