# Supplementary material for: Evidence for positive long- and short-term effects of vaccinations against COVID-19 in wearable sensor metrics
Source: PNAS Nexus. 2023 Jul 25;2(7):pgad223. doi: 10.1093/pnasnexus/pgad223 (PMC10368316; doi:10.1093/pnasnexus/pgad223)
Supplement: pgad223_Supplementary_Data [file pgad223_supplementary_data.pdf]

Supporting Information for

Evidence for positive long- and short-term effects of vaccinations against COVID-19 in wearable sensor metrics

Marc Wiedermann, Annika H. Rose, Benjamin F. Maier, Jakob J. Kolb, David Hinrichs and Dirk Brockmann

Corresponding author: Marc Wiedermann
E-mail: wiedermannm@rki.de

This PDF file includes:

Supporting text
Figs. S1 to S18
Table S1
SI References

Supporting Information Text

General functioning of the Corona Data Donation project

The Corona Data Donation project (Corona-Datenspende-App or CDA) was launched in April 2020 as a smartphone app that connects with consumer-grade smartwatches or fitness trackers and allowed participants to submit basic vital data, such as daily resting heart rate, step count and sleep parameters for pandemic research. Until December 2022 the app was available for download at Google Play, the Apple App Store and Huawei App Gallery.

The general user experience of the app is depicted in Fig. S1. Note that the CDA was only available in German language, but we provide an explanation of the content of each page in the app below. After installation and first opening the CDA participants were greeted by a welcome screen (Fig. S1A) to inform them of the overall purpose of the project and provide some details. Afterwards, users were informed about the project's privacy policy (not shown) that needed to be accepted before being able to continue. Users were then shown their randomly generated pseudonym and a 4-digit pin (not shown) that was needed to, e.g., request deletion or correction of personal data. The next step asked for basic demographic information (Fig. S1B), such as approximate birth year (in 5-year intervals), gender and ZIP-code to foster certain cohort analyses. Afterwards, participants connected their wearables by logging into the website of the specific manufacturer and allowing the CDA to access the data collected by the respective device(s), Fig. S1C. Vital data that was measured after establishing this connection was then transferred to the Robert Koch Institute for research purposes approximately once every night. It is important to note that through this process and for strict data privacy reasons, we were not able to access any data that was collected prior to connecting a device. In later versions of the CDA, users could also choose not to connect any device while still being able to participate. After setting up all desired wearable connections, the users selected which studies that were conducted within the CDA they would like to participate in (not shown).

The concept of a *study* allowed users to define which data to provide on a fine-grained basis. In total, four such studies were part of the CDA. After consenting to participate in the CDA, users automatically enrolled in the first study, the so-called *fever monitor*. Within that study users pseudonymously submitted their wearable-derived vital data for pandemic research. If users did not connect any device, they were still enrolled in that study, but simply did not provide any data. The other three studies contained topic-focused survey modules to complement the collected vital data with additional context: (i) *Tests, symptoms & life situation* contained a single one-off survey and infrequent short update questionnaires on, e.g., approximate COVID-19 test dates and results, symptoms during the disease and general socio-demographics beyond what was surveyed directly after installing the CDA (see above). (ii) *Experience & behaviour in the pandemic* used one-off, weekly and monthly questionnaires to survey, e.g., self-assessed risks, protective behaviour and approximate COVID-19 test results on a rolling basis. (iii) *Long-COVID and pandemic consequences* was the latest addition to the collection of studies and provided monthly questionnaires on long-term health and well-being during the pandemic. For the purpose of our present work, data collected within the *fever monitor*-study and the first two survey modules were evaluated.

Once consented to participate in the desired stud(ies), users were taken to the home screen of the app (Fig. S1D) which displayed a study overview, statistics on the number of completed surveys, the total days of provided vital data, and a list of upcoming questionnaires. Participants enrolled in survey studies received a push notification whenever a new questionnaire was available. Upon opening the notification, users were shown an overview of all open questionnaires (Fig. S1E) and asked to select which one to answer next. Then, an interactive session was started which prompted users to answers a series of topic-focused survey questions (Fig. S1F). Once all questions in the questionnaire were completed, the responses were transferred to the Robert Koch Institute with a timestamp of the completion time.

The steps depicted in Fig. S1A-C were usually only completed upon initial registration, while participants enrolled in survey studies usually frequently came back to the steps shown in Fig. S1D-E.

Distribution of positive PCR-tests over time

As mentioned in the main manuscript most infections in vaccinated individuals took place during the first three waves of the pandemic while breakthrough infections were mostly recorded during the Delta and Omicron waves in late 2021 and 2022, Fig. S2. In fact, the latest infection of an unvaccinated person was reported in June 2021 and only 4 vaccinated individuals report an infection prior to that month.

Influence of variant-specific breakthrough infections on the main results

Vital changes after breakthrough infections with B.1.617.2 compared to unvaccinated individuals. To account for the fact that the analysis in the main manuscript does not discriminate breakthrough infections by the respective variant of concern, we repeat the corresponding analysis only for cases that were reported before December 15, 2021, Fig. S3. During that time only the more severe B.1.617.2 (Delta) Variant was predominant (1).

Recall from the main manuscript that we found significant differences in resting heart rate (RHR) between unvaccinated and vaccinated individuals at a high significance level ($\alpha = 0.01$) at almost all weeks, except the two weeks following a positive PCR-test. Likewise vaccinated individuals differed significantly from the COVID-19 negative control group at weeks -1, 0, 3 and 5-6. In addition, we found that RHR-changes of vaccinated individuals consistently fall below those of unvaccinateds. If we now restrict our analysis to infections in vaccinated individuals that were likely caused by Delta, we find that this general pattern still holds, Fig. S3A. Due to the smaller sample size of Delta infections we do, however, adjust the significance level

to $\alpha = 0.05$. Average RHR-changes for unvaccinated individuals still significantly exceed those of vaccinateds in the week preceding a positive PCR-test, as well as the weeks 2-4, 8 and 10-11 after the test. Hence, the general trends towards lower expected RHR-changes for vaccinated individuals also holds if the analysis is restricted to the Delta variant of concern. This is particular important in the context of our work since Delta is generally considered the variant that causes the most severe cases. Hence, it is reasonable to conclude that an infection of a vaccinated person with Delta is likely still less pronounced with respect to RHR changes than an infection with the B.1.1.7 (Alpha) variant or the wild-type of SARS-CoV-2, albeit the latter two being associated with less severe courses of the disease.

We find similar patterns for physical activity (Fig. S3B), i.e. the weekly averaged number of daily steps taken, even though vaccinated individuals show significantly reduced values for weeks 0-5 (again using a significance level of $\alpha = 0.05$) compared to only the first three weeks after a PCR-test for the entire cohort, cf. Fig. 2B in the main manuscript. Still, activity reduction in unvaccinated individuals takes much longer (up to 12 weeks) to return to normal values, again indicating that vaccines also mitigate risks of long-term activity reduction after an infection with Delta.

Ultimately, sleep duration (Fig. S3C) of vaccinated individuals after an infection with Delta is only significantly increased for the two weeks following a positive PCR-test which is en par with the observations for the entire cohort, compare again Fig. 2C in the main manuscript. Hence, also with respect to this vital type we see no significant qualitative differences in the vaccinated cohort if we restrict our analysis to Delta infections.

Vital changes for breakthrough infections with B.1.617.2 compared to B.1.1.529. We perform an additional analysis to compare the observed vital changes in vaccinated individuals between the two pandemic waves for which respective test dates are available, see also Fig. S2. In particular, we split the vaccinated user cohort into one that reports an infection prior to December 15, 2021 and one that reports a positive PCR-test after that date. We assume that for the former the infection was likely caused by B.1.617.2 (Delta) and for the latter it was caused by B.1.1.529 (Omicron). In analogy to Fig. 2 in the main manuscript and Fig. S3, we compute average changes in RHR, step count and sleep duration for both cohorts, Fig. S4. We further use a two-sided Welch t-test at a significance level of $\alpha = 0.01$ to assess whether the respective averages have to be considered different.

For all three vital signs, we find similar qualitative temporal evolutions as well as magnitudes in the respective changes. Especially for RHR, we find large a similarity in the weeks around a positive PCR-test when comparing breakthrough infections in the two respective cohorts, Fig. S4A. Only from week 2 onward does the return to baseline take a slightly different shape depending on the considered variant. However, at no point in time can the two averages be considered statistically different at a significance level of $\alpha = 0.01$, indicating a likely similar imprint of an infection with either variant of concern on RHR.

Likewise, we find almost the same maximum reduction of $\sim 3,000$ steps per day in the week after a positive PCR-test regardless of the variant that caused the breakthrough infection, Fig. S4B. Moreover, the respective averages only differ significantly during the week of the PCR-test and the second week after, Fig. S4B. A visual inspection of Fig. S4B suggests that the return to baseline takes slightly longer for a breakthrough infection with Delta which aligns with the common observation of a generally milder course of COVID-19 after an Omicron infection (2, 3).

Ultimate, we find again similar patterns across variants when considering average changes in sleep duration, Fig. S4C. Average sleep duration increases by ~ 24 minutes per day for both breakthrough infections with B.1.617.2 or B.1.1.529. A visual inspection again suggests a somewhat slower return to pre-disease values for a Delta infection compared to Omicron. This observation is underlined by a statistically significant difference in the expected sleep duration in the second week after the PCR-test, Fig. S4.

Taken together, we conclude that it is reasonable to combine all recorded breakthrough infections into a single user cohort since differences in vital changes between the two major variants B.1.617.2 and B.1.1.529 are small and hardly significant. Moreover, the analysis presented in Fig. S3 emphasizes that the results presented in the main manuscript do not change substantially on a qualitative level if only breakthrough infections before December 15, 2021 are considered in the vaccinated user cohort. Since B.1.617.2 is considered to be the variant of concern that causes severe courses of COVID-19 most often, our analysis implies that the average vital changes in vaccinated users that suffer from such an infection are still lower than those of unvaccinated individuals that sustained an infection with B 1.1.7. (Alpha) or the wildtype.

Observed time differences between vaccinations and infection

For all recorded breakthrough infections, we compute the approximate time difference between receiving the last vaccination dose and the date of the PCR-test, Fig. S5. Note that vaccination dates are only available with an accuracy of one month and PCR test dates with an accuracy of one week. Hence, we cannot rule out that individuals contracted COVID-19 in the first two weeks after receiving the second vaccine dose, potentially misclassifying their case as a breakthrough infection since the vaccination might not have been fully effective by then. However, only $\sim 2.3\%$ of all breakthrough cases are recorded in the month of the last vaccination dose. Hence, this effect can be considered negligible.

Representativeness of study cohort with respect to age

As already discussed in the main manuscript, our study cohort is not representative of the overall German population. However, a comparison with official census data from 2020[*] reveals that at least the frequencies of the commonly defined age groups

[*] obtained from https://www-genesis.destatis.de/genesis/online

25-39 and 60-64 are well recaptured in our study cohort, Fig. S6. We note a large over-representation of people aged 40-59 and consequentially an under-representation of elderly (age 65 and older) and children/adolescents (age 20 or younger). The latter group is, by definition, mostly excluded from our study since participation is only possible for citizens ages 16 or older. The elderly group is likely under-represented due to a generally lower adoption of new technologies with older people.

References

1. Robert Koch-Institut, SARS-CoV-2 Sequenzdaten aus Deutschland (2022).
2. J Nealon, BJ Cowling, Omicron severity: milder but not mild. *The Lancet* **399**, 412–413 (2022).
3. E Callaway, H Ledford, How bad is omicron? What scientists know so far. *Nature* **600**, 197–199 (2021).

Supplementary Figures

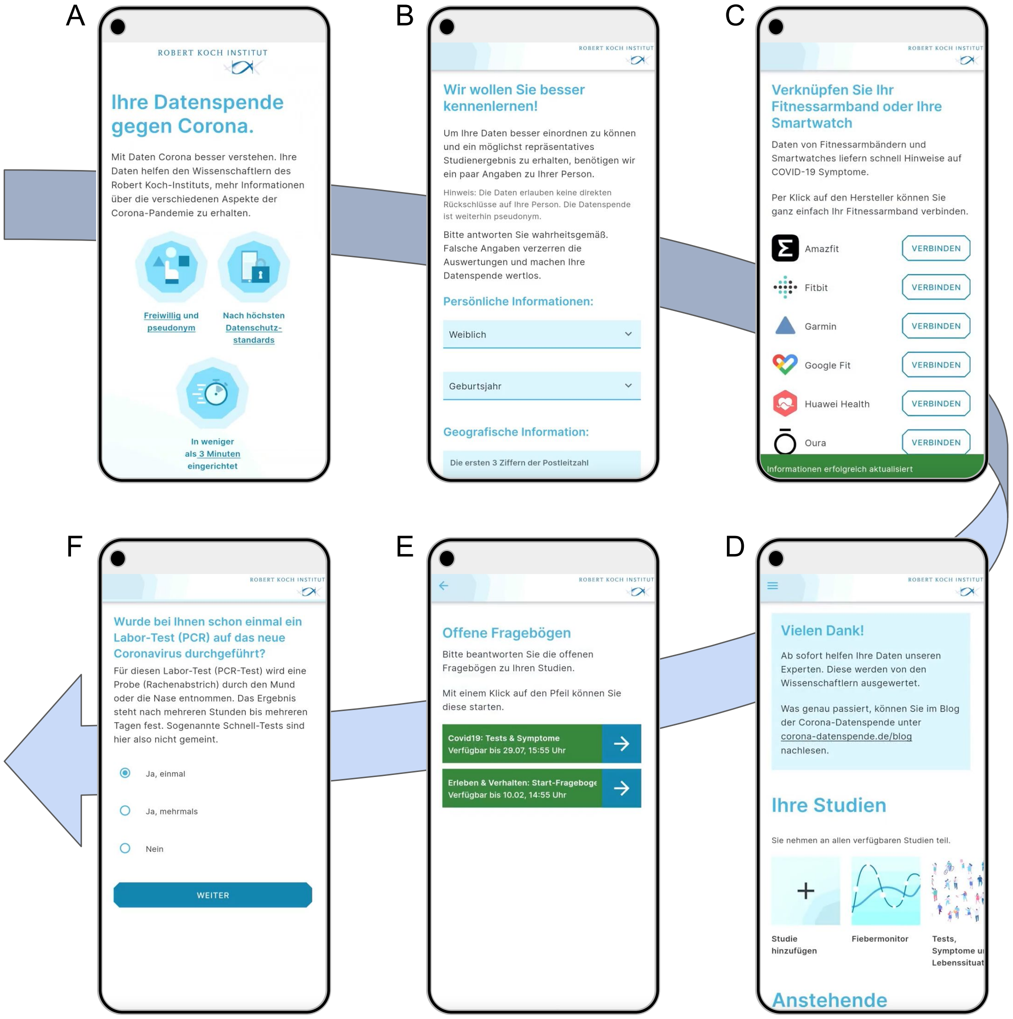

Fig. S1. Participant experience when using the app of the Corona Data Donation project on a smartphone. Note that the app was only available in German language. After installing it from Google Play, Apple App Store or Huawei AppGallery, participants were greeted by a welcome screen (A) with a general introduction to the project. Afterwards, basic demographic information, such as approximate birth year and gender was surveyed (B). Participants then had the possibility to connect the app with their wearable devices (C). From thereon, daily aggregates of resting heart rate, activity and sleep parameters were submitted to the Robert Koch Institute approximately every night. (D) The home screen of the app showed an overview of the studies that each user was enrolled in. Participants taking part in any of the survey studies received a push notification whenever a new questionnaire was available. Upon opening the notification, participants were brought to an overview page of unanswered questionnaires (E). After selecting one of the open questionnaires, an interactive session was started that prompted users to answers a series of topic-focused survey questions (F). Upon completion of a questionnaire, this data was transferred to the Robert Koch Institute for analysis.

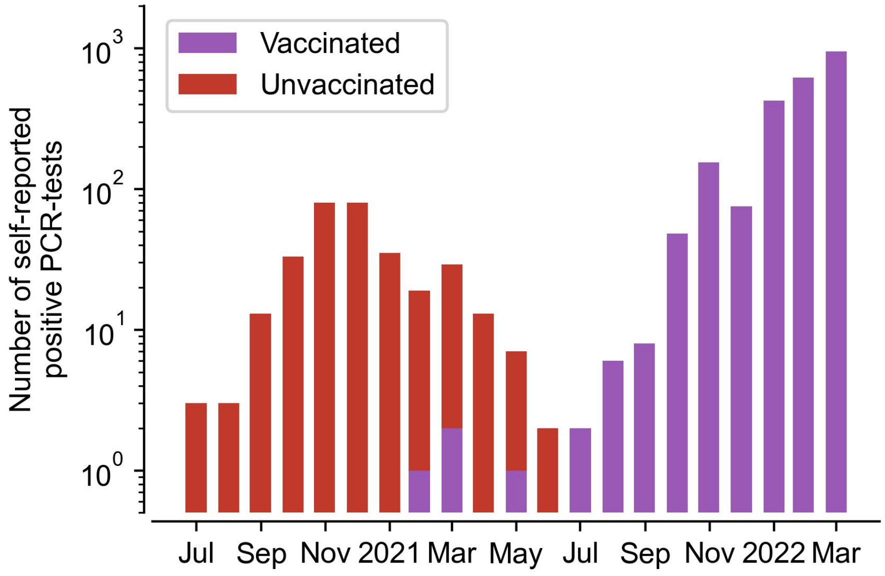

Fig. S2. Absolute count of self-reported positive PCR-tests per month for vaccinated individuals (purple) and unvaccinated individuals (red). Note the logarithmic scale due to an increasing number of positive tests from 2022 onward. Individuals are considered part of the vaccinated cohort when they received at least two doses of mRNA vaccine.

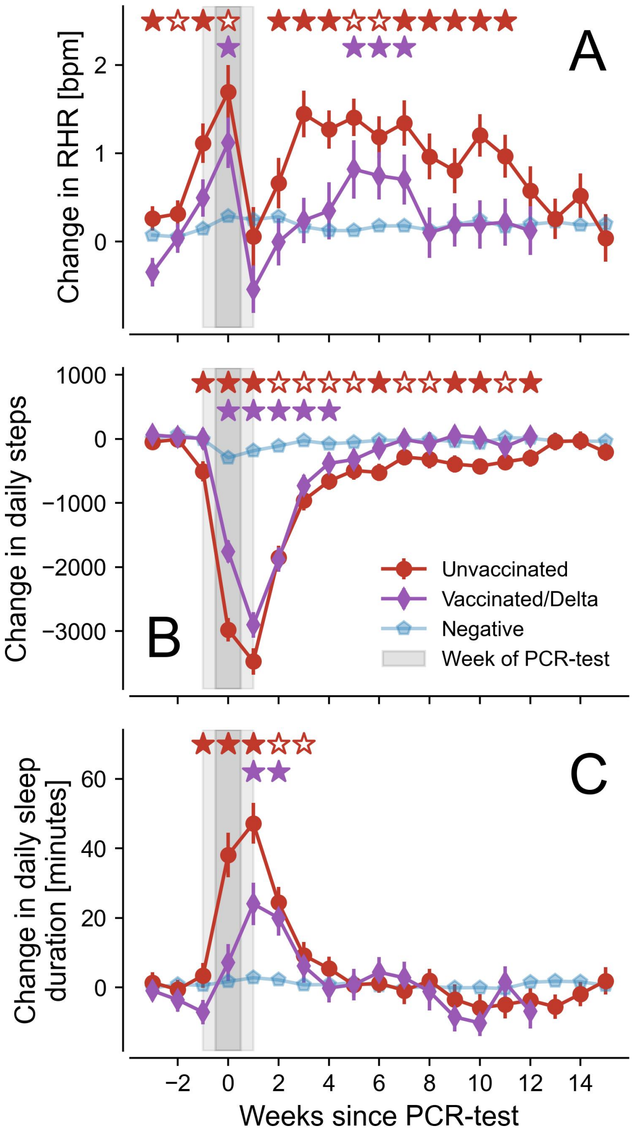

Fig. S3. Same as Fig. 2 in the main manuscript, but only considering breakthrough infections that took place before December 15, 2021, i.e., most likely caused by the B.1.1.7 (Delta) variant. Filled (empty) red asterisks indicate periods where the average vital change of unvaccinated individuals is stronger than that of vaccinated (negative) individuals using a one-sided Welch t-test and a significance level of $\alpha = 0.05$. Purple asterisks indicate significant differences between vaccinated and COVID-19 negative individuals. Individuals are considered part of the vaccinated cohort when they received at least two doses of mRNA vaccine.

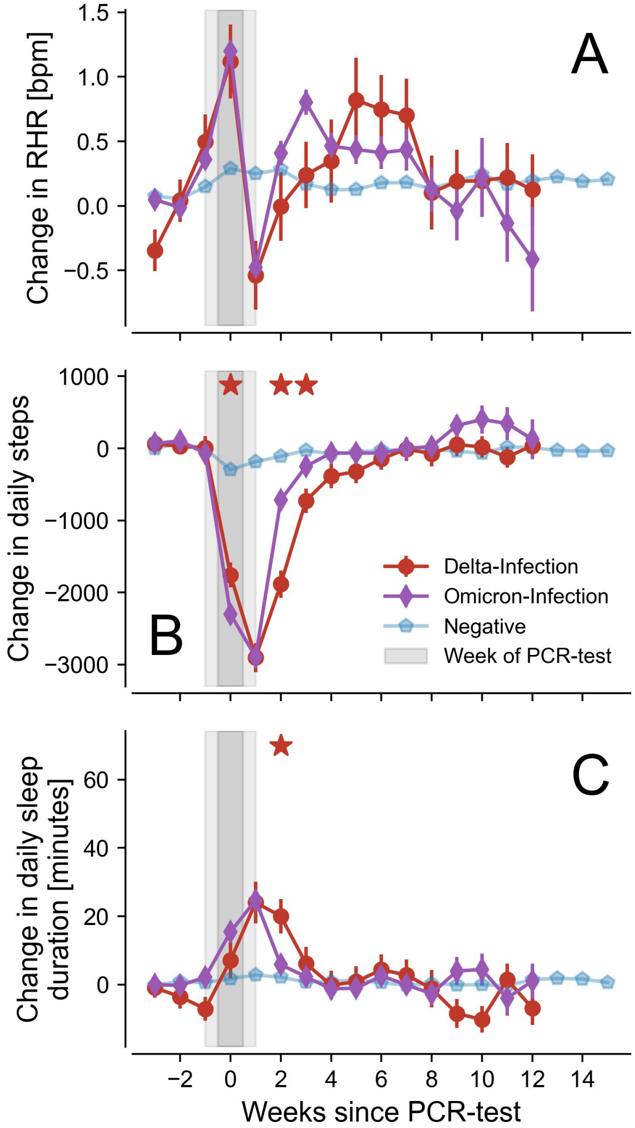

Fig. S4. Changes in RHR, activity, and sleep duration in two vaccinated user cohorts with recorded infections before December 15, 2021 (red) and after (purple), as well as negative controls (blue). Changes are measured relative to the two months preceding the test. Errors bars indicate standard error. Red stars indicate periods where the average vital changes in the two vaccinated cohorts are statistically different using a two-sided Welch t-test and a significance level of $\alpha = 0.01$. Individuals are considered part of the vaccinated cohort when they received at least two doses of mRNA vaccine.

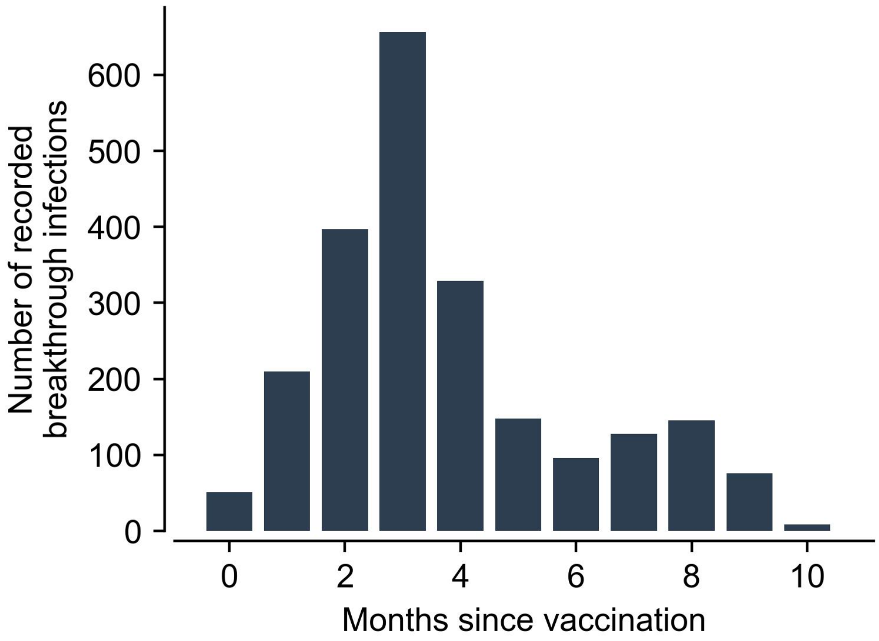

Fig. S5. Time difference in months between a confirmed infection with SARS-CoV-2 and receival of the last vaccination dose, i.e., the second or third dose depending on status. A time difference of 0 months indicates that infection took place less than 30 days after receiving the last vaccination.

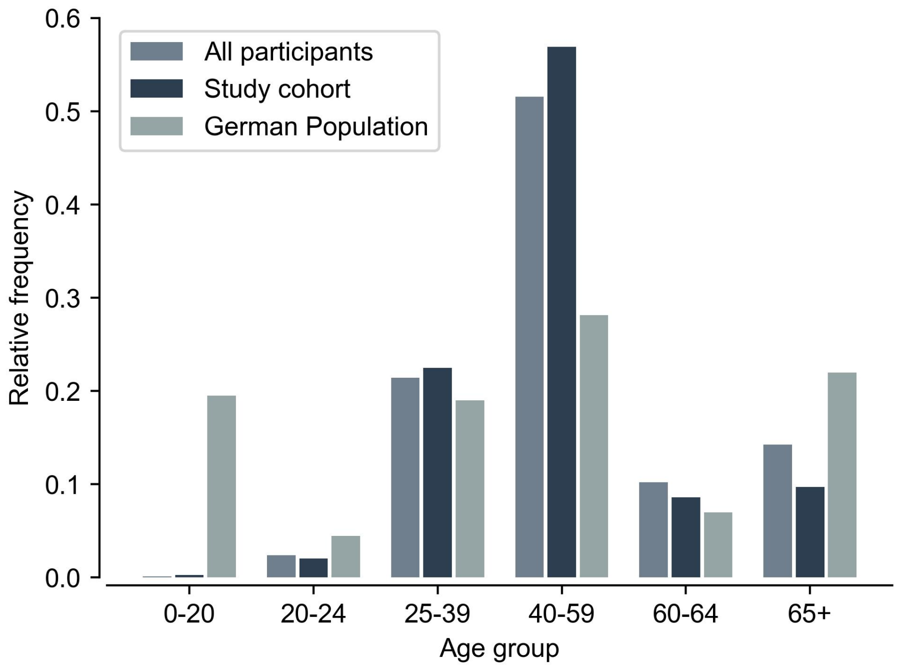

Fig. S6. Relative age frequencies in (i) our study cohort, (ii) all participants in the Corona Data Donation project that provide basic demographic information (237,091 out of 535,557 individuals), and (iii) the overall German population.

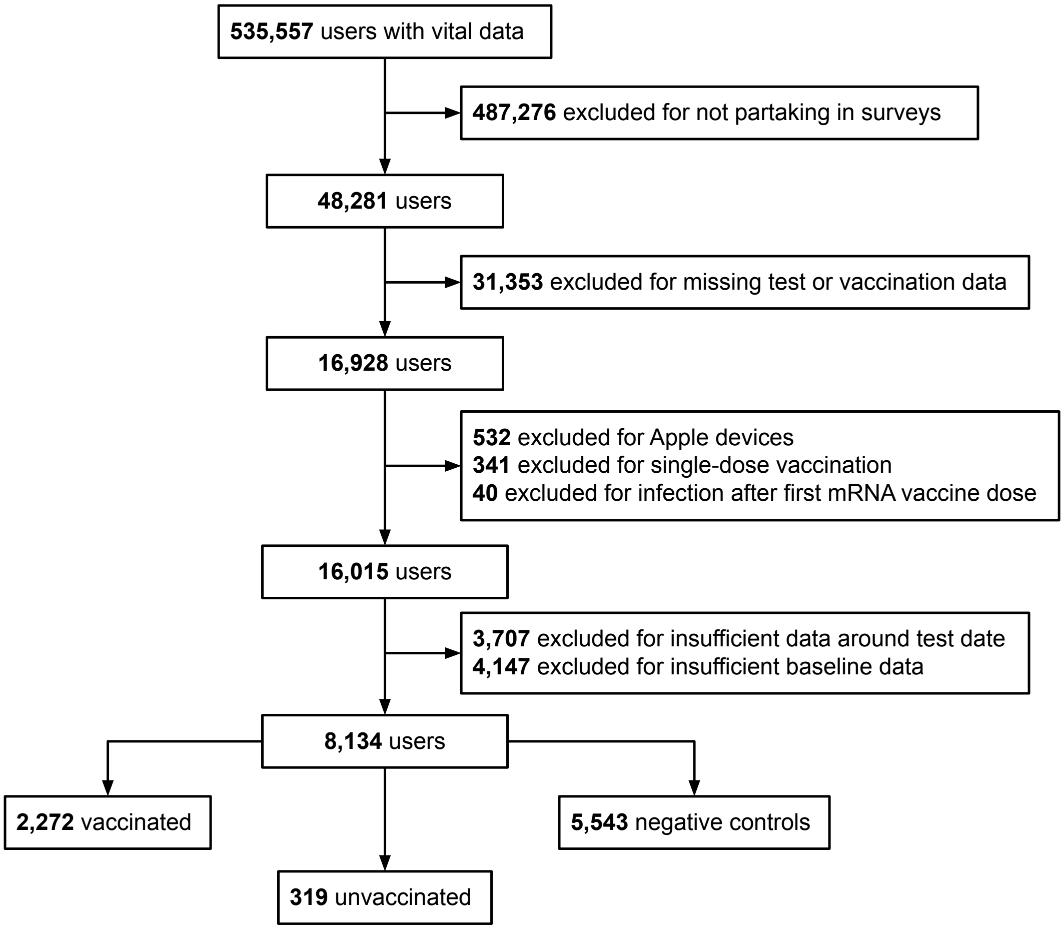

Fig. S7. Cohort diagram of the study group.

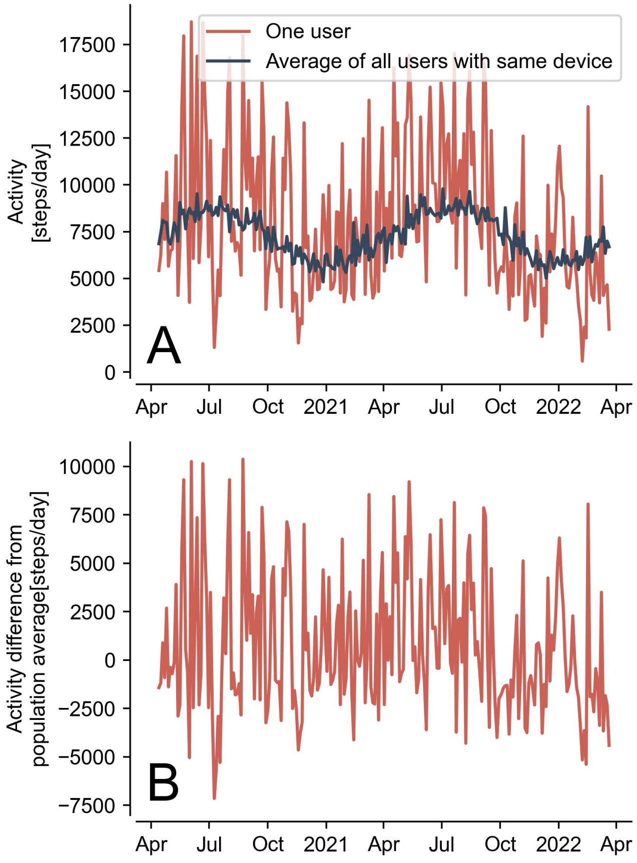

Fig. S8. Example of transforming an activity time series measured in steps/day into per-user anomalies that are measured against a population-wide baseline. (A) Exemplary daily activity of a single participant (red) as well as the corresponding average daily activity of all individuals with the same wearable device (black). The latter shows a periodic signal with increased activity in summer and lowered activity in winter. Such external influences might artificially cause indications of decreased step counts after a potential PCR-test taken in summer or even increased activity after a PCR-test taken in winter. We therefore subtract the population-wide average from each user's time series data and obtain per-user anomalies where at least such periodic signals are absent (B). Changes in vital data are then more likely observed due to specific characteristics of the individual, rather than due to external environmental influences.

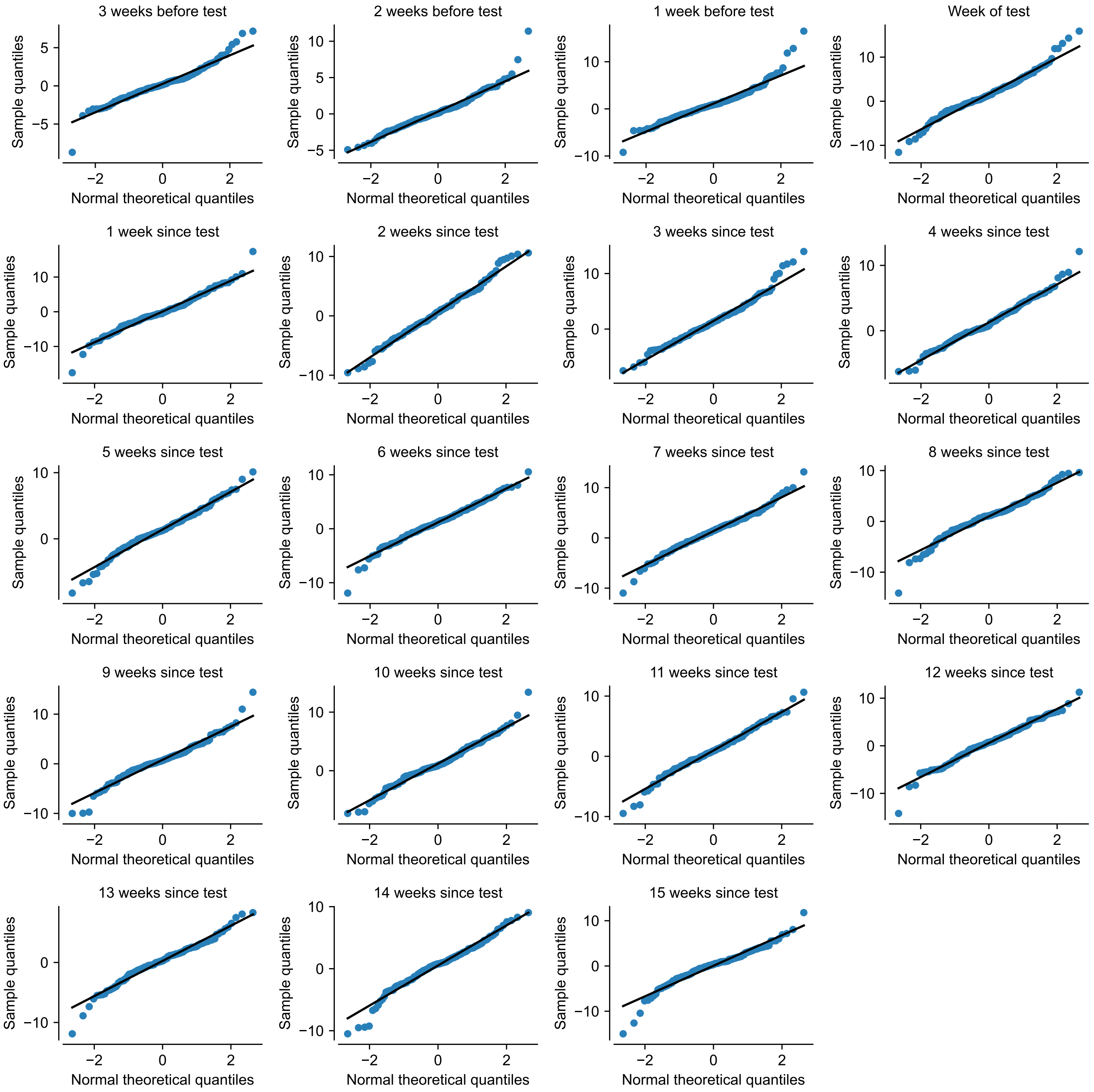

Fig. S9. Quantile-quantile diagram for changes in average resting heart rate (RHR) between 3 weeks prior and 15 weeks after taking a negative COVID-19 PCR-test. Sample quantiles (in bpm) are compared to theoretically expected quantiles from a normal distribution.

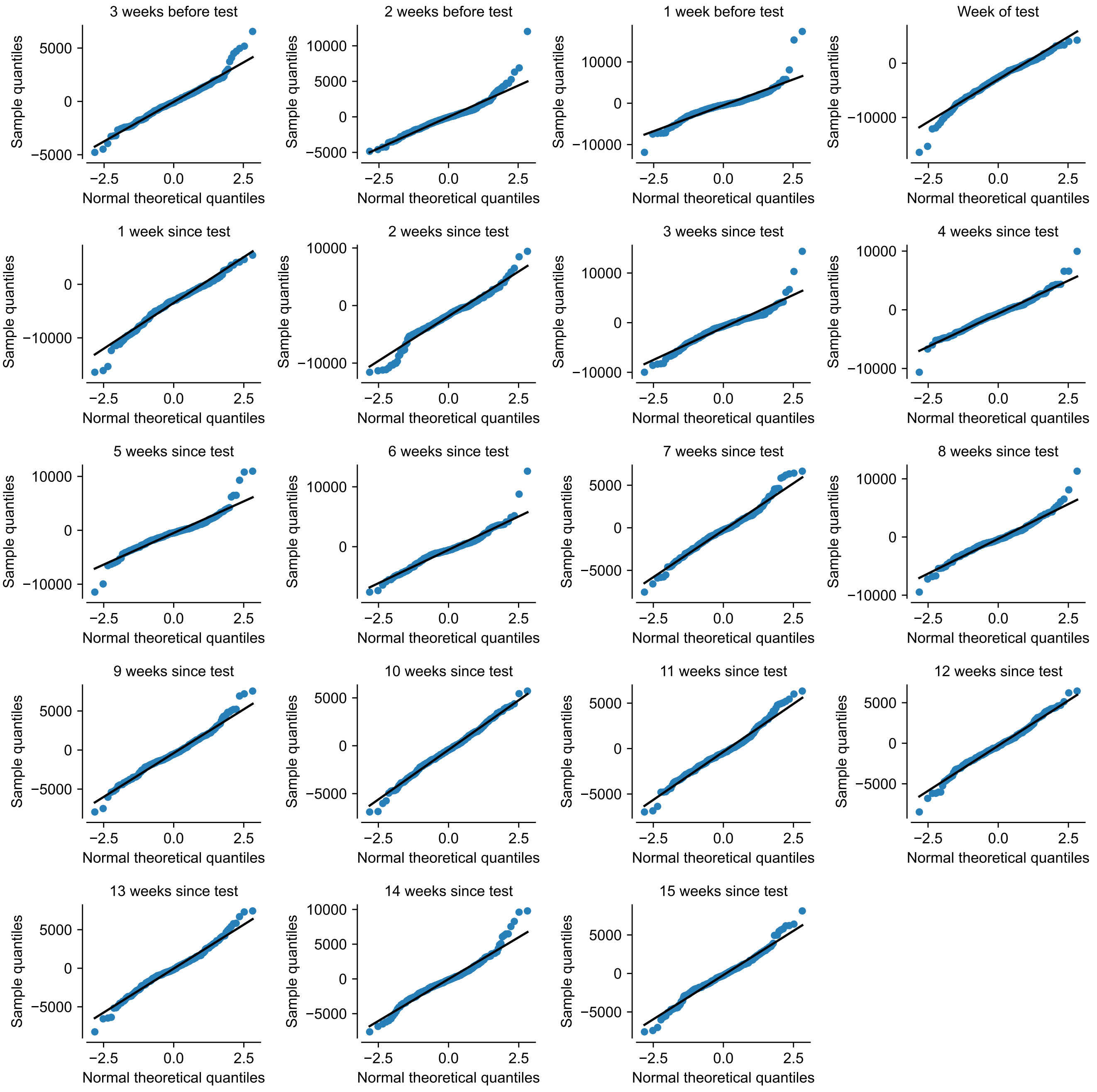

Fig. S10. Same as Fig. S9, but for changes in average daily activity. Sample quantiles are measured in terms of step count.

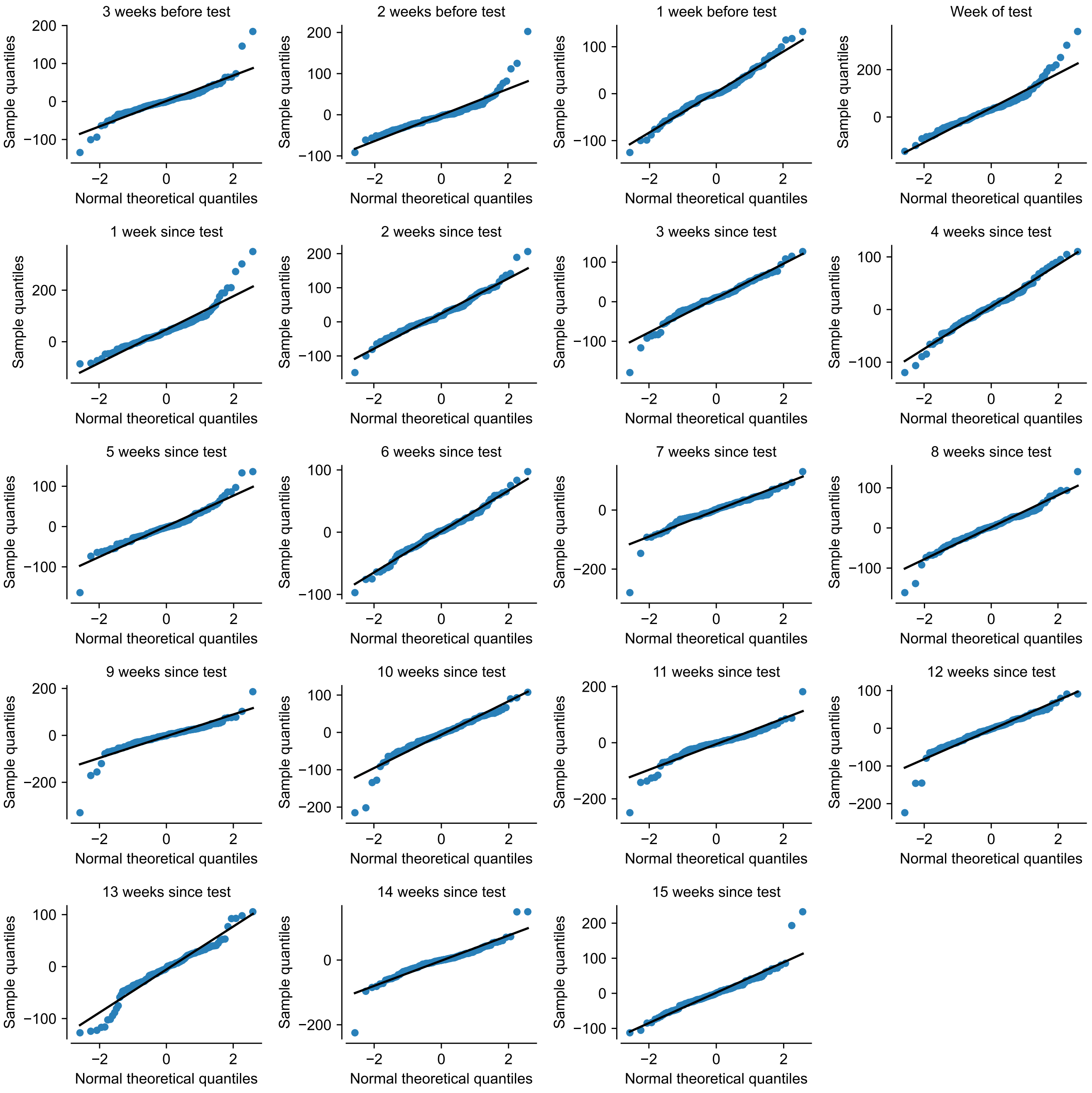

Fig. S11. Same as Fig. S9, but for changes in average sleep duration (in minutes).

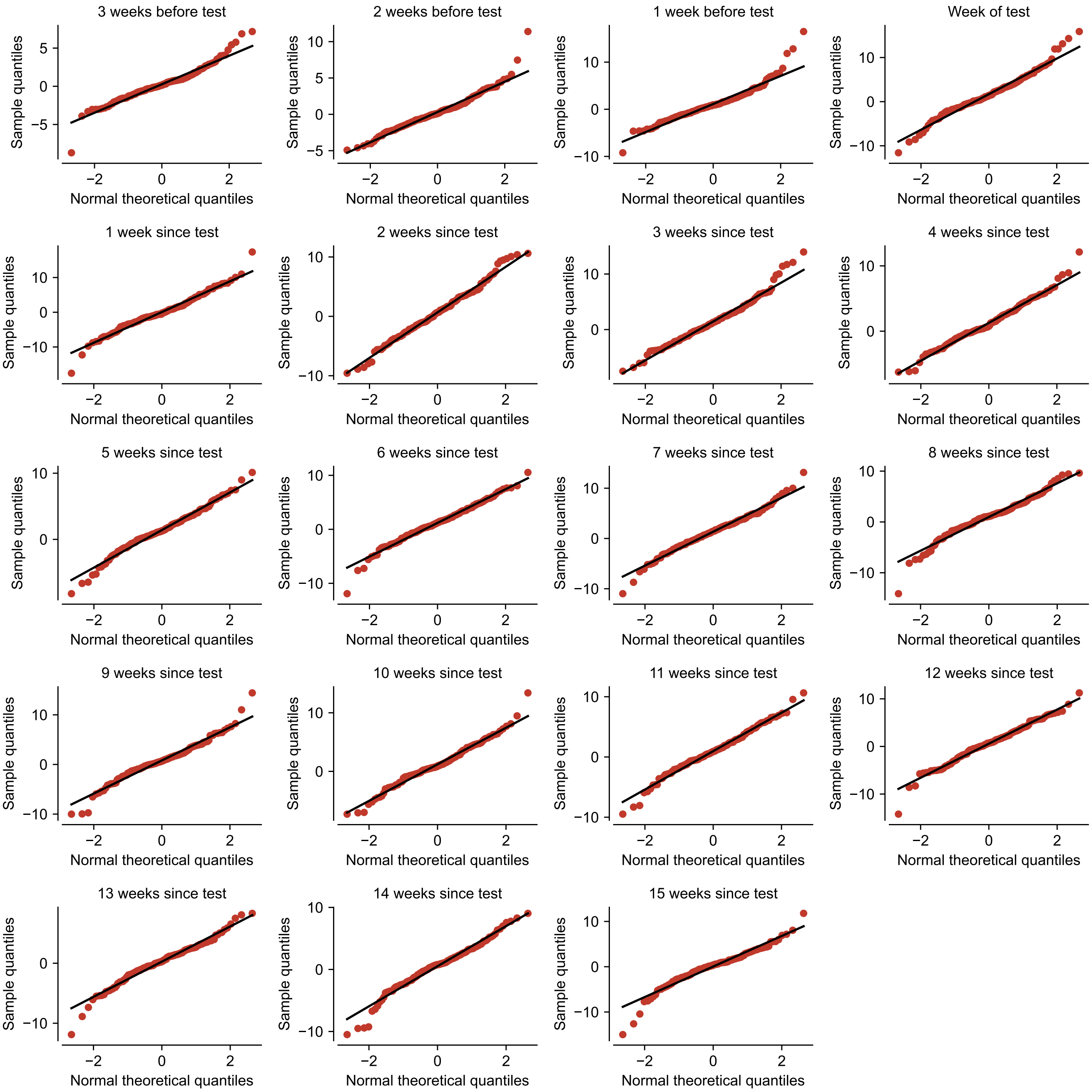

Fig. S12. Same as Fig. S9 but for participants that report a positive COVID-19 PCR-test and were unvaccinated by the time of infection.

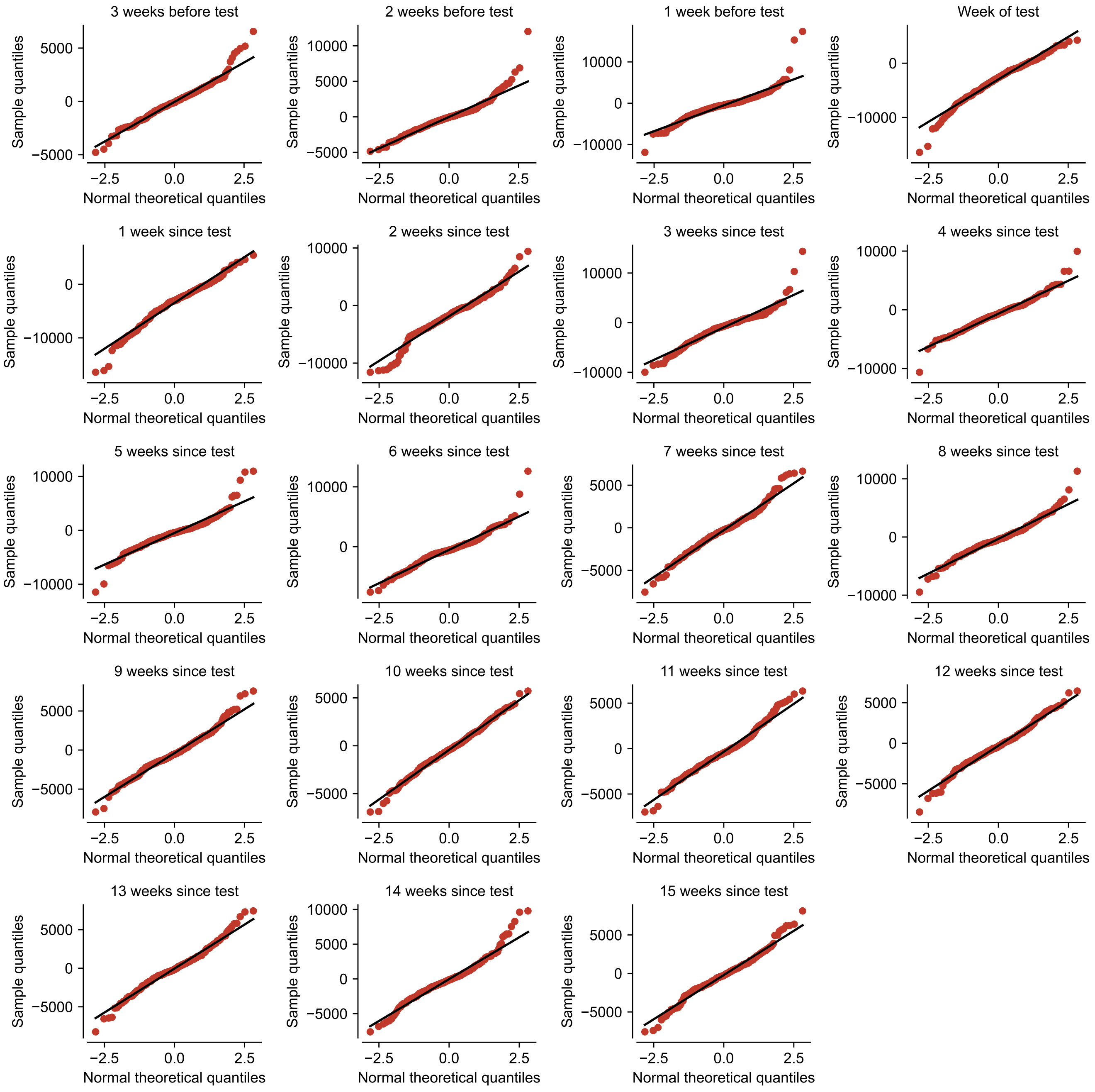

Fig. S13. Same as Fig. S12, but for changes in average daily activity. Sample quantiles are measured in terms of step count.

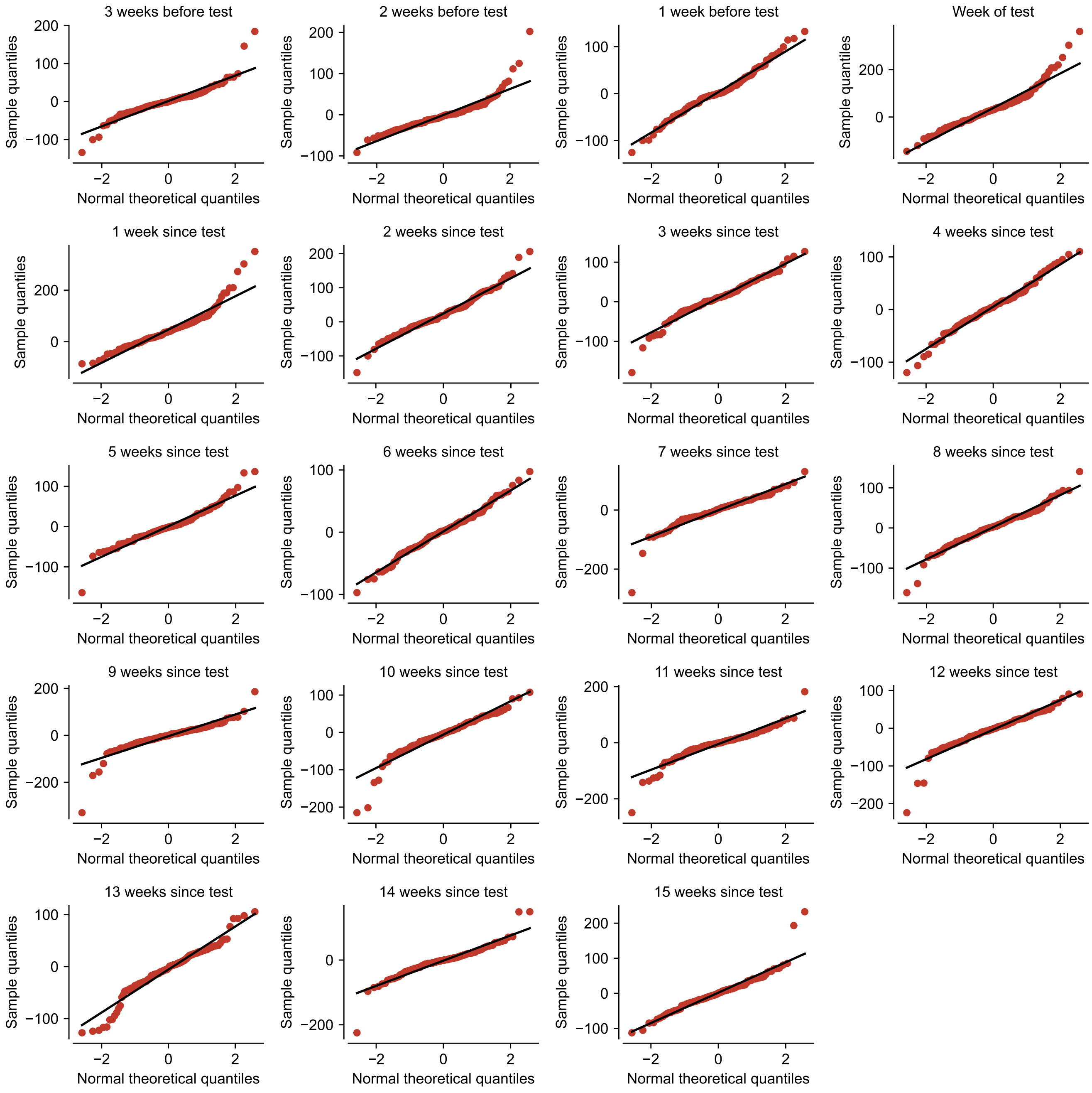

Fig. S14. Same as Fig. S12, but for changes in average sleep duration (in minutes).

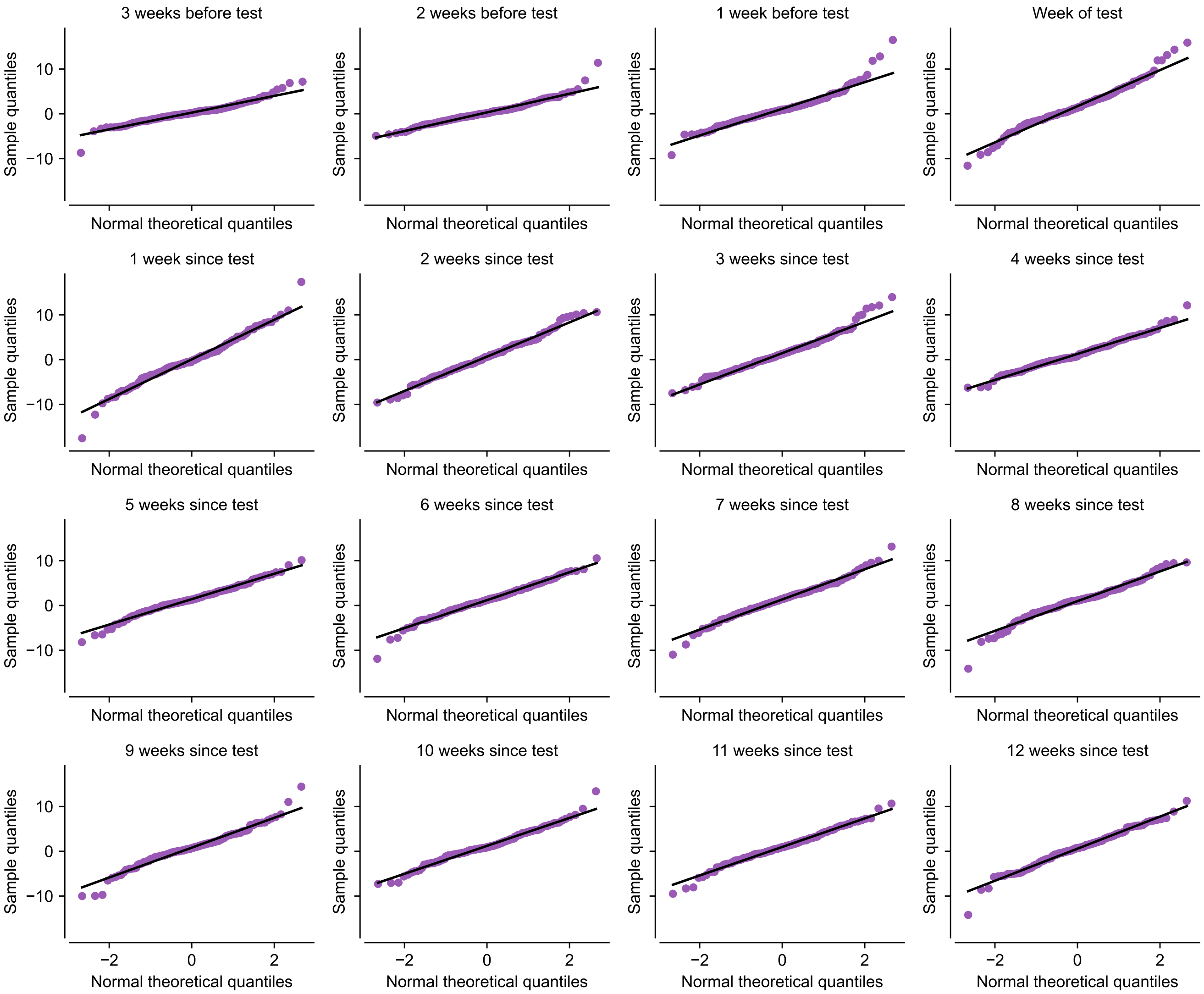

Fig. S15. Same as Fig. S9 but for participants that report a positive COVID-19 PCR-test and were vaccinated by the time of infection. In alignment with Fig. 2 of the main manuscript, results are only shown for the first twelve weeks after a positive PCR-test due to insufficient data for later time periods.

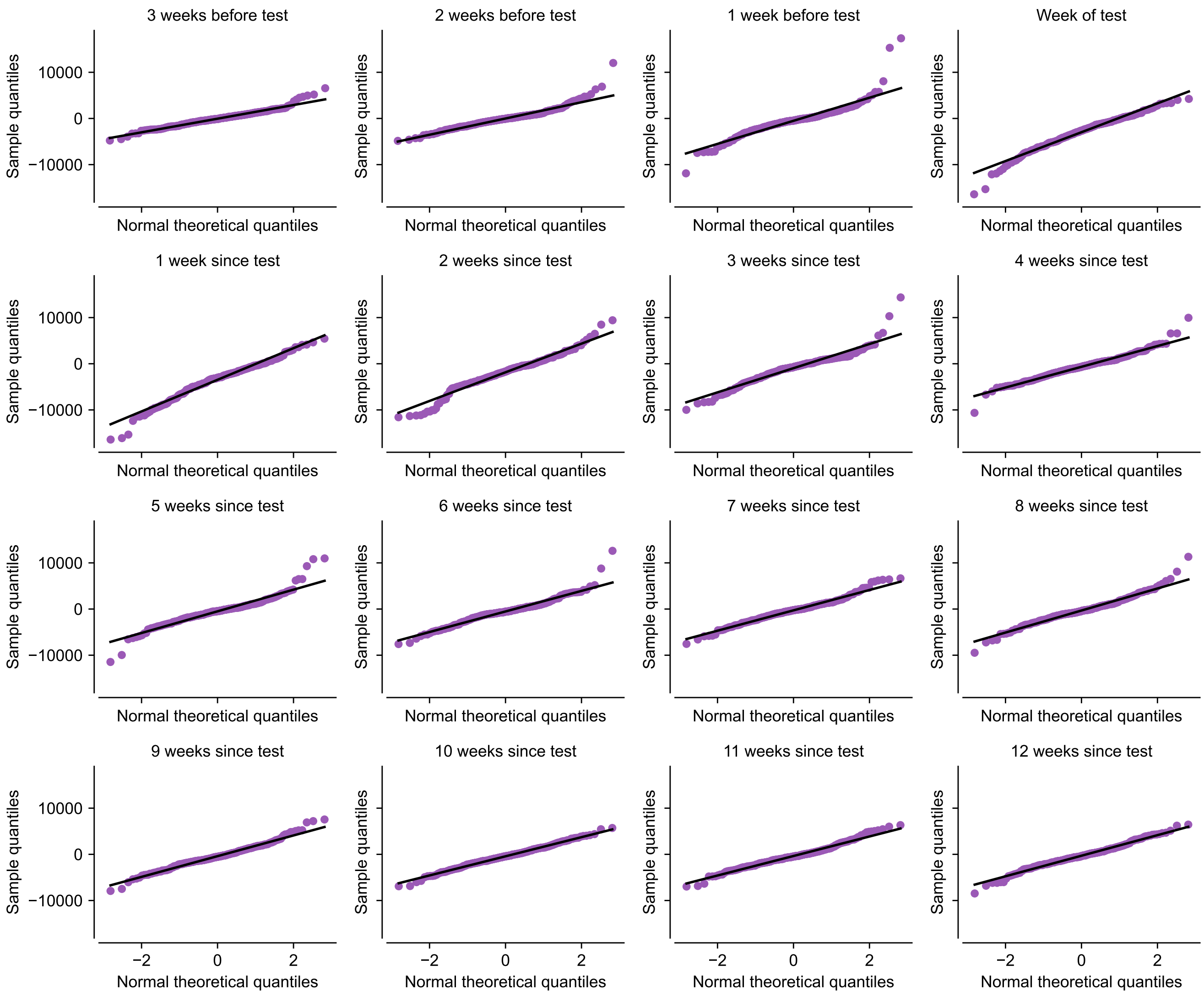

Fig. S16. Same as Fig. S15, but for changes in average daily activity. Sample quantiles are measured in terms of step count.

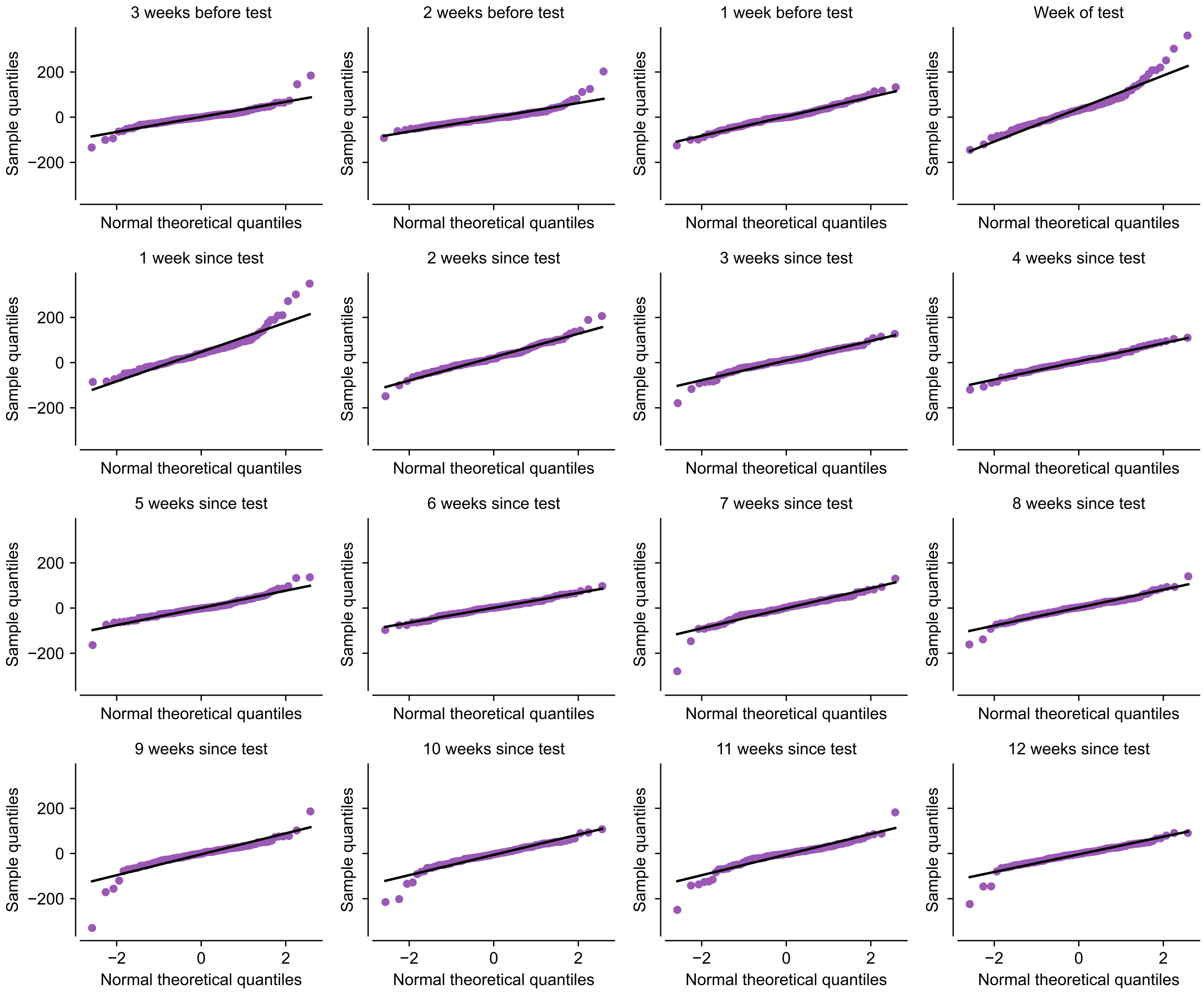

Fig. S17. Same as Fig. S15, but for changes in average sleep duration.

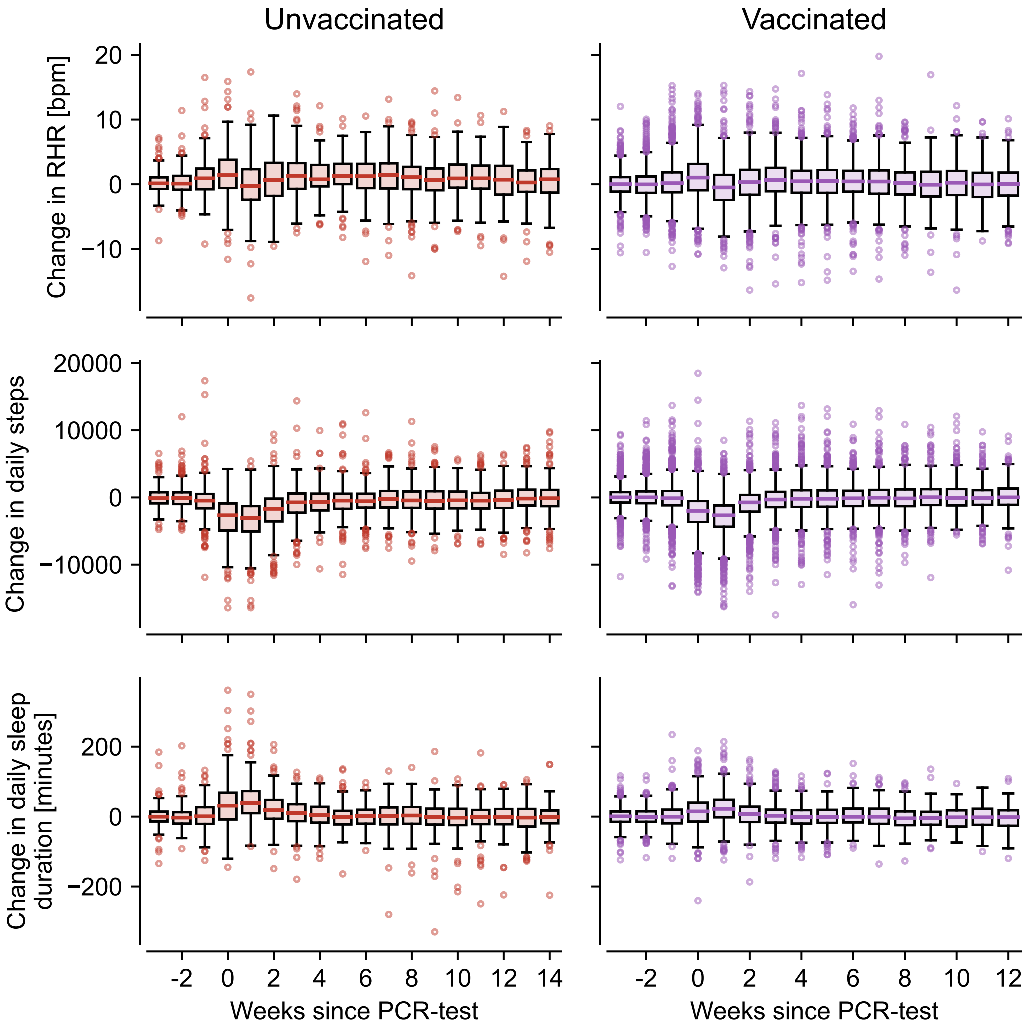

Fig. S18. Distribution of changes in resting heart rate (RHR), activity, and sleep duration in unvaccinated infected (red) and vaccinated infected (purple) individuals around a positive COVID-19 PCR-test (analogous to Fig. 2 in the main manuscript). Solid horizontal lines indicate the median, boxes indidate the interquartile range (IQR), whiskers denote 1.5 times the IQR and scatter points denote measurements outside the range indicated by the whiskers, i.e., outliers. The indicated median values show the same trend as discussed in the main manuscript. Moreover, we find no notable (large set of) outliers that might artificially skew the depicted averages in Fig. 2 of the main manuscript towards increased or decreased values.

	Total in Data Donation project
Female	86825 (36.62%)
Male	150001 (63.27%)
Other	265 (0.11%)
Age (mean)	51.82yr
Age (std)	13.45yr

Table S1. Number of users per gender as well as mean and standard deviation of age for the 237,091 participants in the Corona Data Donation project that provide such basic demographic information. The numbers are comparable to those of the study cohort, cf. Tab. 1 in the main manuscript.
